# Antimicrobial Activity of Some Steroidal Hydrazones

**DOI:** 10.3390/molecules28031167

**Published:** 2023-01-24

**Authors:** Maia Merlani, Nanuli Nadaraia, Lela Amiranashvili, Anthi Petrou, Athina Geronikaki, Ana Ciric, Jasmina Glamoclija, Tamara Carevic, Marina Sokovic

**Affiliations:** 1 TSMU I. Kutateladze Institute of Pharmacochemistry, Tbilisi 0159, Georgia; 2 School of Pharmacy, Aristotle University of Thessaloniki, 54124 Thessaloniki, Greece; 3 Mycological Laboratory, Department of Plant Physiology, Institute for Biological Research “Siniša Stankovi’c”, University of Belgrade, 11060 Beograd, Serbia

**Keywords:** hydrazones, ketosteroids, antimicrobial activity

## Abstract

Twelve steroid based hydrazones were in silico evaluated using computer program PASS as antimicrobial agents. The experimental evaluation revealed that all compounds have low to moderate antibacterial activity against all bacteria tested, except for *B. cereus* with MIC at a range of 0.37–3.00 mg/mL and MBC at 0.75–6.00 mg/mL. The most potent appeared to be compound **11** with MIC/MBC of 0.75/1.5 mg/mL, respectively. The evaluation of antibacterial activity against three resistant strains MRSA, *E. coli* and *P. aeruginosa* demonstrated superior activity of compounds against MRSA compared with ampicillin, which did not show bacteriostatic or bactericidal activities. All compounds exhibited good antifungal activity with MIC of 0.37–1.50 mg/mL and MFC of 1.50–3.00 mg/mL, but with different sensitivity against fungi tested. According to docking studies, 14-alpha demethylase inhibition may be responsible for antifungal activity. Two compounds were evaluated for their antibiofilm activity. Finally, drug-likeness and docking prediction were performed.

## 1. Introduction

The development of new antimicrobial agents is still attracting the interest of medicinal chemists since the resistance of bacterial pathogen strains is a major problem.

One of the reasons for the fast multiplication of bacteria is their ability to exchange genes with each other, leading to the development of resistance. On the other hand, the interest in the discovery of new antimicrobial agents is because during the past 30+ years, the FDA has approved only two new antimicrobial drugs: linezolid and daptomycin.

Despite the fact that many compounds have been synthesized and tested, their clinical use has been restricted due to the high risk of toxicity and pharmacokinetic deficiencies. Thus, the scientists have directed their efforts at developing novel approaches to antimicrobial therapy, aiming to overcome the resistance problem [1,2,3,4]. Another big problem is biofilm formation, which plays a crucial role in bacterial infection and antimicrobial resistance. There is increasing proof that cells in biofilms, on a biotic or abiotic surface, are 1000-fold more resistant to conventional drugs than planktonic cells [5,6]. The problem is that upon being established, biofilms become difficult to eliminate and as a result, chronic and persistent infections [7] appear. As reported in the literature [8,9], one of the main Gram-positive pathogens causing biofilm-associated infections is *Staphylococcus aureus.* Thus, another need is for the development of new agents that are able to inhibit *S. aureus* biofilm formation.

**Figure 1:**
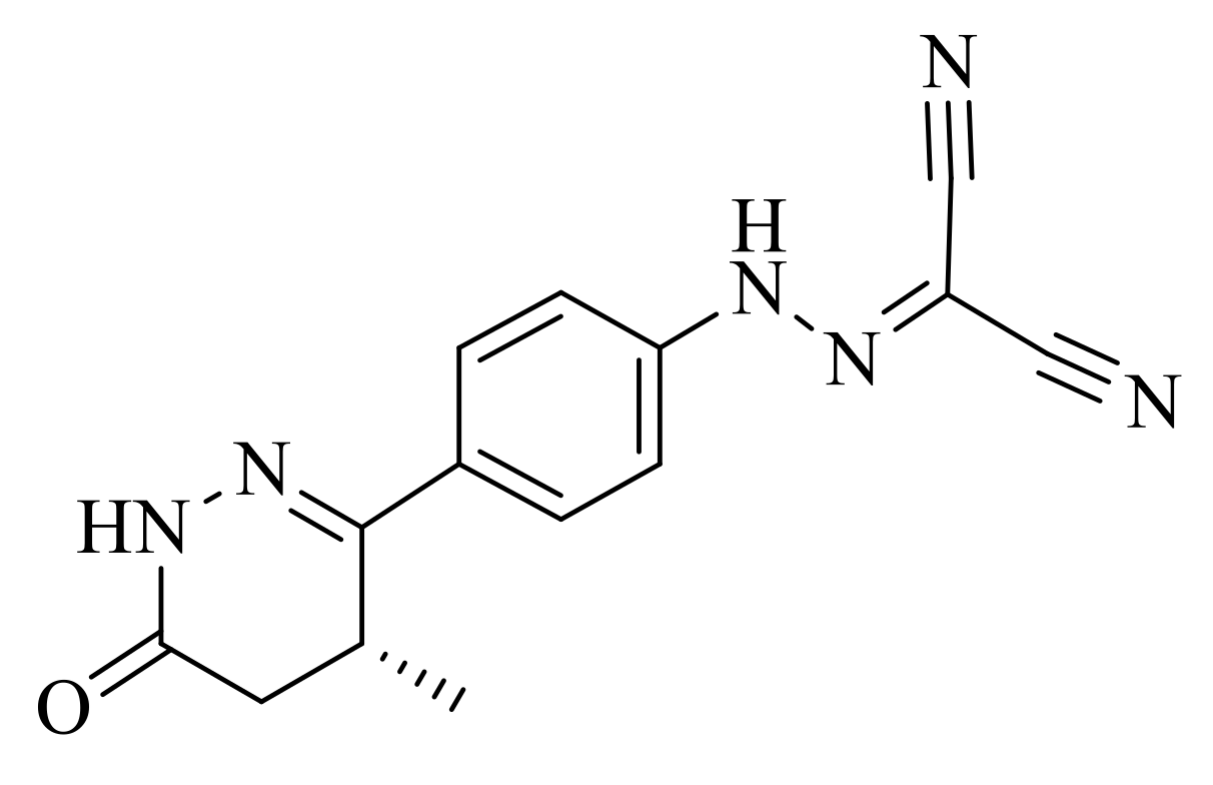
Approved by FDA drug.

Hydrazones of different chemical classes possess diverse biological and pharmacological properties such as antimicrobial, anti-inflammatory, analgesic, antifungal, anti-tubercular, antiviral, anticancer, antiplatelet, antimalarial, anticonvulsant, cardio protective, anthelmintic, antiprotozoal, anti-trypanosomal, anti-schistosomiasis etc. [10,11,12]. Hydrazones contain two connected nitrogen atoms of different nature and a C-N double bond that is conjugated with a lone electron pair of the terminal nitrogen atom. These structural fragments are mainly responsible for the physical and chemical properties of hydrazones. The combination of thehydrazono group with other functional groups leads to compounds with a unique physical and chemical character [13]. It is noteworthy that there is an approved FDA drug with a hydrazone scaffold, namely levosimendan, a calcium sensitizer used in the management of acutely decompensated congestive heart failure (Figure 1).

On the other hand, steroidal compounds are a class of bioactive substances playing a major role in living organisms with a wide representation in the natural world. Steroidal derivatives attracted the interests of scientists, especially medicinal chemists, due to their wide range of biological activities [10,13,14,15]. They are known to possess antimicrobial [16,17], antioxidant [17] and anticancer [17] activities. In the last few decades, the efforts have concentrated on rational modification of steroid molecules due to their lower toxicity, vulnerability to multi-drug resistance and high bioavailability to penetrate the cell wall and to be linked to nuclear and membrane receptors. Vollaro et al. [18] reported the investigation of the in vitro effect of pregnadiene-11-hydroxy-16α,17α-epoxy-3,20-dione-1 (PYED-1) on biofilm formation.

Nowadays, a number of steroidal hydrazone derivatives have been developed and evaluated for their antimicrobial activity [19,20,21,22,23,24]. Among these hydrazones are some 5α-steroidal derivatives of the androstane and pregnane series with different functional groups.

Encouraged by these observations, and based on our previous work [25,26,27], herein we report the synthesis of two novel 5α-steroidal hydrazones and the evaluation of antimicrobial activity of newly and earlier synthesized compounds.

Thus, the purpose of our study was in silico and biological evaluation of the antimicrobial potential of twelve steroidal hydrazino derivatives, including action on the resistant strains.

## 2. Results and Discussion

### 2.1. Chemistry

In the continuation of our research on new bioactive N-containing 5α-steroids, ten steroidal hydrazine derivatives, that we synthesized earlier on the basis of steroidal ketones [25,26,27,28,29], and two new compounds were prepared and evaluated for their antibacterial and antifungal actions.

**Scheme 1:**
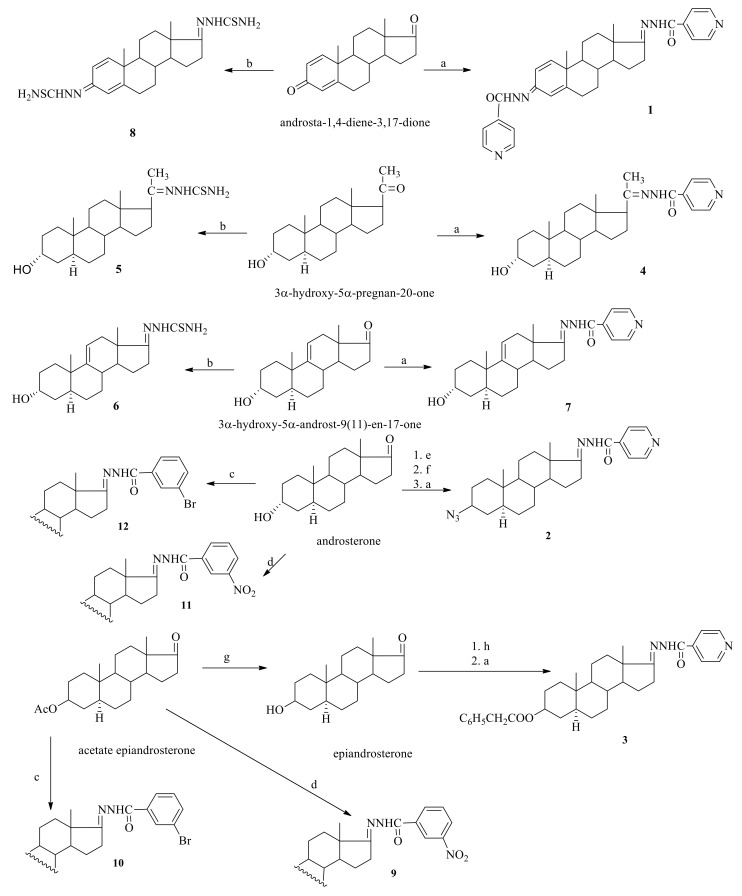
Synthetic route of substances (**1**–**12**): (a) isoniazide, EtOH, CH_3_COOH, reflux 2 h; (b) thiosemicarbazide, EtOH, CH_3_COOH, 2 h; (c) *m*-bromobenzohydrazide, EtOH, CH_3_COOH; (d) *m*-nitrobenzohydrazide, EtOH, CH_3_COOH, reflux, 5 h; (e) TsCl, pyridine, 0 °C, 20 h; (f) NaN_3_, DMF, 100 °C, 5 h; (g) NaOH, MeOH, reflux 30 min; (h) ClCOCH_2_C_6_H_5_, pyridine, benzene, reflux 6 h.

Isonicotinoylhydrazones **1**–**4** and **7** were synthesized from corresponding ketones androsta-1,4-diene-3,17-dione, and rosterone, epiandrosterone, allopregnanolone, 3α-hydroxy-5α-androst-9(11)-en-17-one, by refluxing with hydrazide of isonicotinic acid in ethanol, respectively [25]. Thiosemicarbazones **5**, **6** and **8** were obtained from allopregnanolone, 3α-hydroxy-5α-androst-9(11)-en-17-oneandandrosta-1,4-diene-3,17-dione by refluxing with thiosemicarbazide in ethanol, respectively [25]. *m*-Bromobenzoylhydrazones **10** [26] and **12** were synthesized from acetate epiandrosterone and androsterone, respectively, by refluxing with *m*-bromobenzohydrazide. *m*-Nitro hydrazones **9** [26] and **11** [29] were synthesized similarly by refluxing the corresponding ketone with *m*-nitrobenzohydrazide. The synthesis of all these compounds is presented in Scheme 1.

### 2.2. PASS Predictions

PASS prediction of antimicrobial activities was performed for previously synthesized compounds (**1**, **3**–**11**), as well as for two new designed ones. The antibacterial activity was predicted only for two compounds with Pa values in the range 0.164–0.313, and antifungal activity for almost all compounds with Pa values in the range 0.143–0.470. The calculated Pa values for all compounds were less than 0.5, indicating their relative novelty compared to the structures of the compounds from the PASS training set [30]. This may be proof that the studied compounds have some features dissimilar from those of well-known antimicrobial agents, which may indicate their innovative potential.

### 2.3. Biological Evaluation

#### 2.3.1. Antibacterial Activity

Synthesized compounds were tested for their antibacterial activity against a panel of nine bacteria species, using the microdilution method for the determination of minimal inhibitory and minimal bactericidal concentrations (MIC and MBC, respectively). As reference drugs, ampicillin and streptomycin were used. The antibacterial activity of tested compounds (Table 1) in general was low to moderate, except in some cases where it was good, with MIC ranging from 0.37 to 3.00 mg/mL and MBC at 0.75–9.00 mg/mL, presented in Table 1. The order of activity can be presented as follows: **11** = **12** > **4** = **5** > **3** > **1** > **6** > **7** > **10** > **8** > **9** > **2**. Compound **11** appeared to be the most potent among those tested, with MIC and MBC of 0.75/1.5 mg/mL, respectively, but less than for both reference drugs. The most sensitive bacterium was found to be *B. cereus*, whereas *S. aureus* was the most resistant one.

The structure–activity relationship studies revealed that the presence of 3-nitrobenzohydrazide at position 17 of 3α-hydroxy-5α-androstan-17-one **11** is beneficial for antibacterial activity. The replacement of the nitro group in the benzene ring by Br led to compound **12** havingthe same good influence as the previous one, on activity. The replacement of the substituted benzene ring by isonicotinoylhydrazide, and 3α-hydroxy-5α-androstan-17-one by 3α-hydroxy-5α-pregnan-20-one, resulted in compound **4** with slightly lower activity. It is interesting to notice that the presence of thethiosemicarbazide substituent at position 20 (**5**) of the steroid ring, in place of isonicotinoylhydrazide (**4**) as the substituent in position 20, exhibited the same activity as compound **4**. Replacement of hydroxy group in position 3 by phenylacetoxy (**3**) decreased the antibacterial activity more, while replacement by theazide group (**2**) was detrimental.

The evaluation of antibacterial activity of these compounds against three resistant strains, MRSA, *E. coli* and *P. aeruginosa* revealed that compounds were more potent against MRSA than ampicillin, which did not show bacteriostatic or bactericidal activity, while against the two other resistant strains, it did not show bactericidal activity. The order of activity of the tested compounds against resistant strains can be presented as **6** = **7** > **1** > **3** > **4** > **11** = **12** > **5** > **8** > **10** > **9** > **2**, with compounds **6** and **7** being the most potent (MIC/MBC at 0.75–1.50 mg/mL and 1.50–3.00 mg/mL, respectively). It should be mentioned that compounds **3**–**5**, **8**–**12** did not show any activity against the resistant *E. coli* strain. It is interesting to notice that compounds **6** and **7** were more potent against resistant *E. coli* than *E. coli* strains, while the opposite was observed for compounds **1** and **2**. On the other hand, compounds **1**,**3**,**6**,**7** and **8** exhibited better activity against *P. aeruginosa* and against resistant *P. aeruginosa*, while compounds **2**,**4**,**10**–**12** demonstrated the same activity against both of these strains. In general, our compounds showed better activity against the resistant *P. aeruginosa* strain than against two other resistant strains, being less potent than the reference drug streptomycin.

In the case of structure–activity relationship studies against resistant strains, it was found that the presence of athiosemicarbazide substituent (**6**) as well as anisonicotinoylhydrazide one (**7**) in position 17 of 3α-hydroxy-5α-androst-9(11)-en-17-one was favorable for the activity against resistant strains.

Finally, it should be mentioned that compounds tested have different behavior against ATCC and resistant strains. The only common behavior against both strains was observed for compound **2**, which demonstrated a negative effect on antibacterial activity in both cases.

#### 2.3.2. Antifungal Activity

Compounds were also tested for their antifungal activity against six fungal strains using the microdilution method, and ketoconazole as well as bifonazole were used as reference drugs. The results are presented in Table 2. In general, compounds showed moderate to good activity with MIC and MFC in the range of 0.37–3.00 mg/mL and 0.50–6.00 mg/mL, respectively. The order of activity of tested compounds can be presented as follows: **7 = 8 > 3 > 1 = 9 > 12 > 11 > 6 > 10 > 5 > 2 > 4**. The best activity was achieved for 3α-hydroxy-5α-androst-9(11)-en-17-one isonicotinoylhydrazone (**7**), with MIC/MFC of 0.37/0/75 mg/mL, respectively, as well as for compound **8**. The lowest antifungal effect was observed for compound **4**, with an MIC ranging from 0.75 to 3.00 mg/mL and MFC from 1.5 to 6.0 mg/mL. It should be noticed that compounds **7** and **8** showed the best potential against all fungi tested (MIC at 0.37 mg/mL), while compound **3** demonstrated the same effect against all fungi except for *C. albicans*. On the other hand, compounds **9** and **12** also showed the same good activity with previous ones against *A. fumigatus, T. viride, C. albicans* and *A. fumigatus, A. niger, T. viride*, respectively. Ketoconazole exhibited antifungal potential at MIC in the range 0.2–1.0 mg/mL and MFC of 0.3–2.0 mg/mL, while bifonazole at MIC 0.10–0.20 mg/mL and MFC at 0.2–0.3 mg/mL, respectively. All compounds showed higher activity than ketoconazole (MIC/MFC of 1.0/1.5 mg/mL) against *T. viride*, the most sensitive fungal. However, it is more important that almost all compounds, except for **11** and **12**, were more potent than ketoconazole against *C. albicans—*the most resistant to our compounds and the deathliest fungal, responsible together with filaments fungal *A. fumigatus* for 85–90% of deaths.

According to the structure–activity relationship studies, the presence of isonicotinoylhydrazide (**7**) in position 17 of 3α-hydroxy-5α-androst-9(11)-en-17-one core and dithiosemicarbazide of androst-1,4-dien-3,17-dione moiety (**8**) have a positive influence on antifungal activity. Replacement of 3α-hydroxy-5α-androst-9(11)-en-17-one core by 3β -phenylacetoxy-5α-androstan-17-one and introduction to position 17 isonicotinoylhydrazide substituent led to compound **3** havingdecreased activity, which decreased more by introduction of a 3β-azido-5α-androstan-17-one moiety (**2**) instead of 3β-phenylacetoxy-5α-androstan-17-one (**3**). The same influence on antifungal activity resulted from the presence of *m*-nitrobenzoylhydrazide in 3β-acetoxy-5α-androstan-17-one core. The replacement of the 3α-hydroxy-5α-androst-9(11)-en-17-one core of compound **7** by 3α-hydroxy-5α-pregnan-20-one (**4**) and thiosemicarbazide substituent at position 20 by isonicotinoylhydrazide (**4**) weredetrimental for antifungal activity. In general, 3α-hydroxy-5α**-**pregnan-20-one isonicotynoylhydrazone **4** and thiosemicarbazone **5** as well the 3β-azido-5α-androstan-17-one isonicotinoylhydrazone **2** were not favorable for antifungal activity.

#### 2.3.3. Inhibition of Biofilm Formation

**Figure 2:**
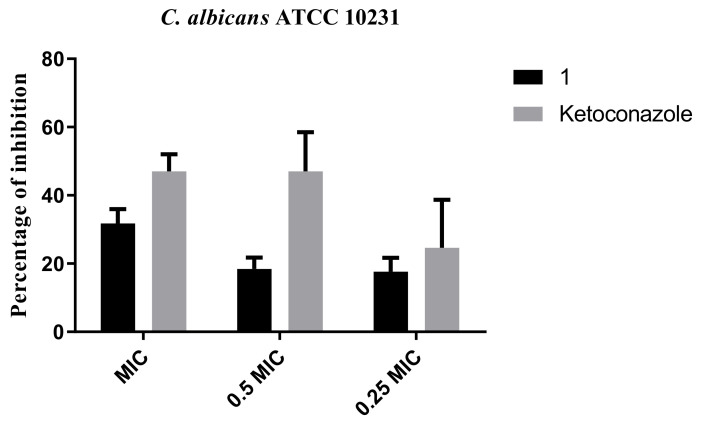
Percentages of inhibition of *C. albicans* ATCC 10231 biofilm formation by compound **1** and Ketoconazole.

**Figure 3:**
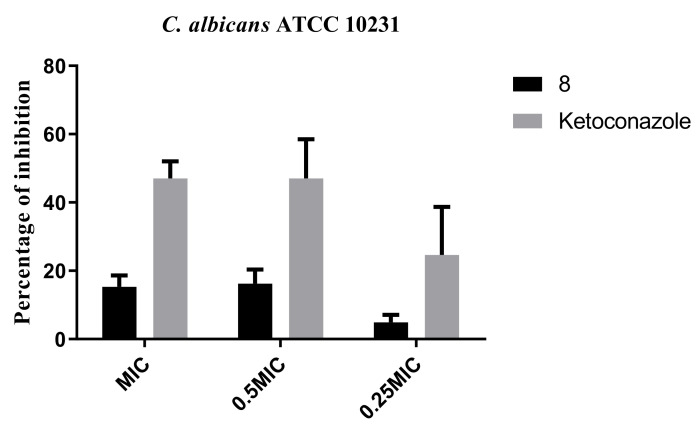
Percentages of inhibition of *C. albicans* ATCC 10231 biofilm formation by compound **8** and Ketoconazole.

After the observation of the antifungal activities of compounds, antibiofilm activities were assessed. We observed that compounds **1** and **8** possessed higher antifungal activity against *C. albicans* and all tested micro fungi than other used compounds. The strain used for the antibiofilm assay was *C. albicans.* Incubation with compounds **1** and **8** hasreduced the ability of *C. albicans* (Figure 2 and Figure 3) to attach to the surface and begin the process of biofilm formation. A concentration equal to the previously determined MIC has reduced the biofilm biomass by 33% and 15% for compounds **1** and **8**, respectively. When applied in 0.5 and 0.25 MIC concentrations of compound **1**, inhibition percentages were almost the same, about 18% (Figure 3). The reference drug, Ketoconazole, possessed better biofilm activity than the compounds, reducing the biofilm biomass by 50%, 47% and 25% for MIC concentrations 0.5 MIC and 0.25 MIC, respectively (Figure 2).

Even twice as low concentrations (0.5 MIC) of compound **8** limited the biofilm forming ability and induced more than 16% inhibition in *C. albicans*. The impact on the fungal biofilm was less profound and the 0.25 MIC concentration of **8** was able to reduce the biofilm formation byless than 5% (Figure 3).

### 2.4. Docking to Antifungal Targets

In order to investigate the possible mechanism of antifungal activity of compounds, all of them along with the reference drug ketoconazole were docked to lanosterol 14α-demethylase of *C. albicans* and DNA topoisomerase IV. The results are presented in Table 3.

**Figure 4:**
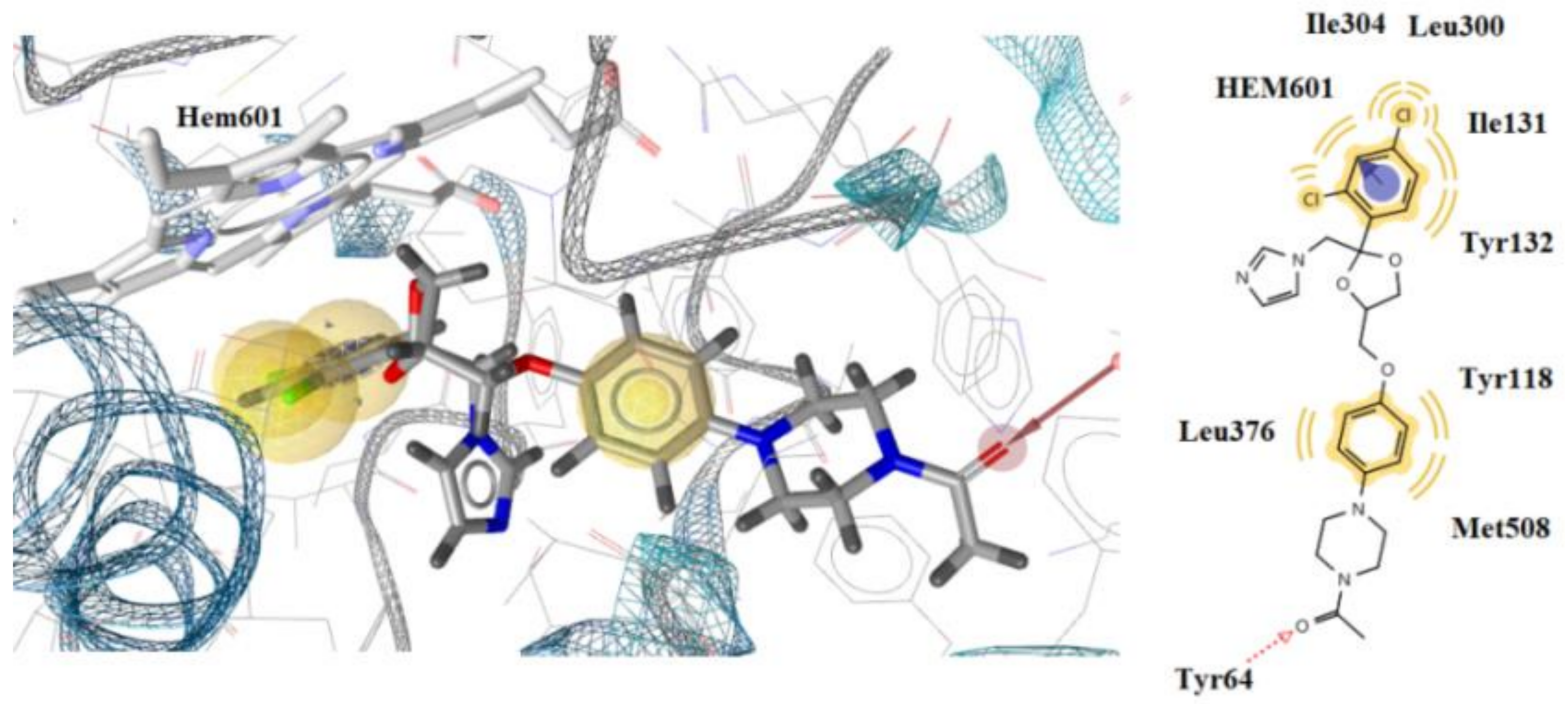
Docked conformation of ketoconazole in lanosterol 14α-demethylase of *C*. *albicans* (CYP51_ca_).

**Figure 5:**
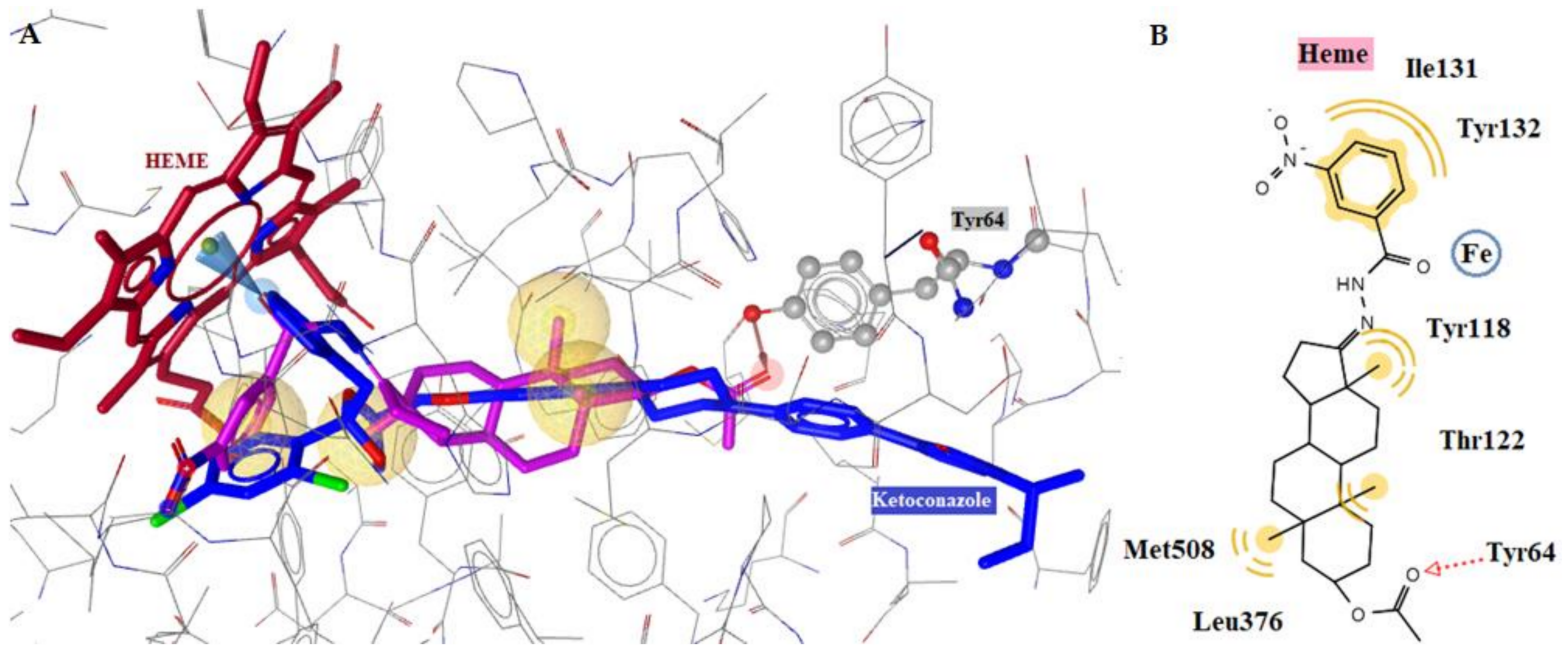
(**A**) Superposition of compound **8** (magenta) and ketoconazole (blue) in lanosterol 14α-demethylase of *C*. *albicans* (CYP51_ca_). (**B**) Docked conformation of the most active compound **8** in lanosterol 14α-demethylase of *C*. *albicans* (CYP51_ca_). Red dotted arrows indicate H-bond, blue arrows aromatic interactions and yellow spheres hydrophobic interactions.

Based on docking studies, all compounds bind to the CYP51 Ca enzyme similarly to the reference drug ketoconazole (Figure 4). The most active compound **8** binds to the Fe of the heme and interacts hydrophobically and aromatically with the heme. Additionally, compound **8** forms a hydrogen bond between the oxygen atom of the C=O group and the side-chain hydrogen of Tyr64. Hydrophobic interactions were also detected between residues I Tyr118, Thr122, Ile131, Tyr132, Leu376, Met508 and the compound (Figure 5). Ketoconazole also forms aromatic and hydrophobic interactions with the heme group. It has been shown, however, that compound **8** forms a more stable complex with the enzyme, possibly due to its interaction with heme’s iron. It is likely that this is the reason why this compound has a better antifungal effect than ketoconazole.

**Figure 6:**
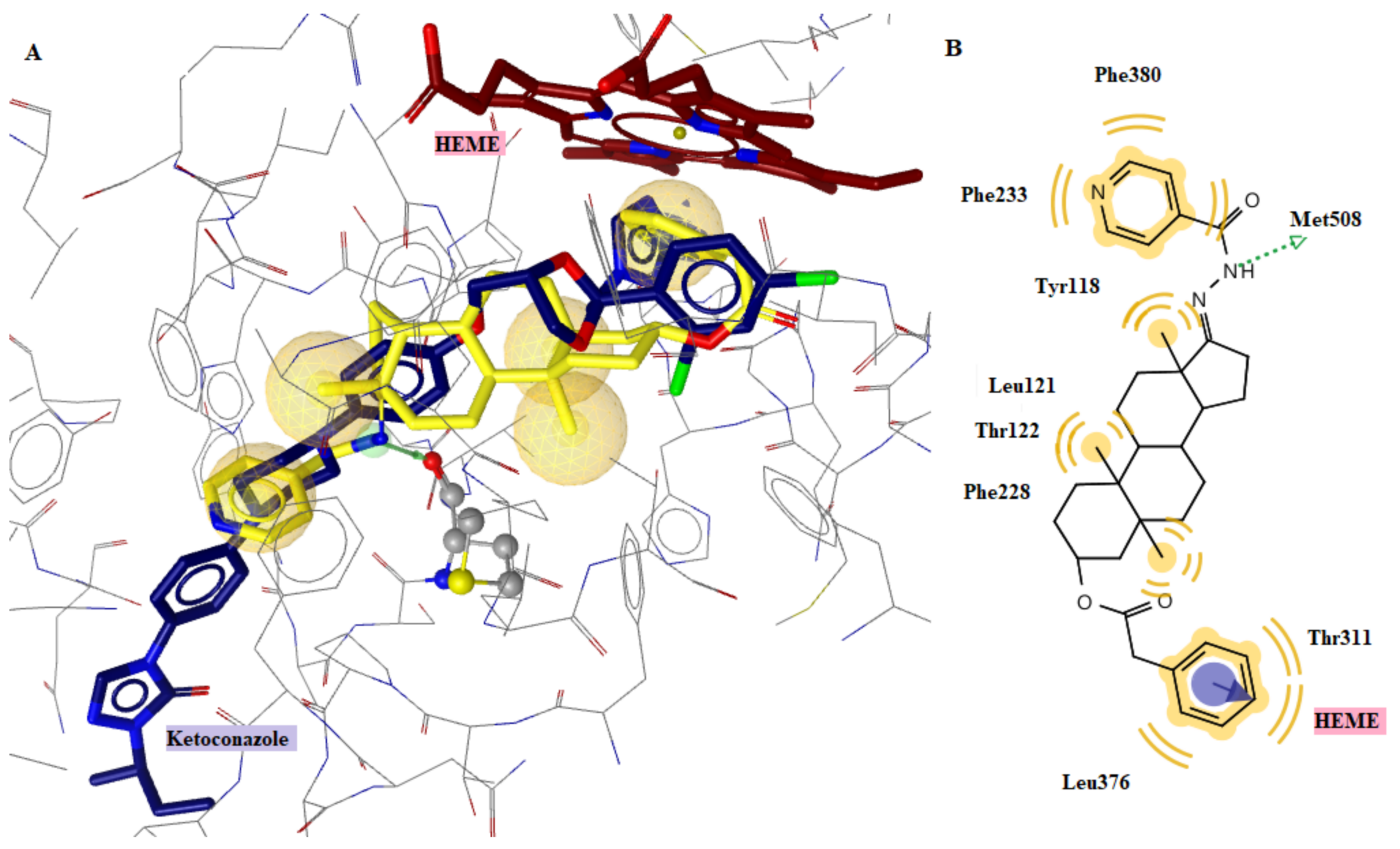
(**A**) Superposition of compound **3** (yellow) and ketoconazole (blue) in lanosterol 14α-demethylase of *C*. *albicans* (CYP51_ca_). (**B**) Docked conformation of compound **3** in lanosterol 14α-demethylase of *C*. *albicans* (CYP51_ca_). Red dotted arrows indicate H-bond, blue arrows aromatic interactions and yellow spheres hydrophobic interactions.

The superposition of compound **3** and ketoconazole (Figure 6) explains its good antifungal activity. Similar to ketoconazole, compound **3** inserts the binding site of the enzyme, forming an additional hydrogen bond with residue Met508. In addition, it exhibits the same hydrophobic and aromatic interactions with the heme group as ketoconazole, which explains its good inhibition profile.

### 2.5. Drug-Likeness

**Figure 7:**
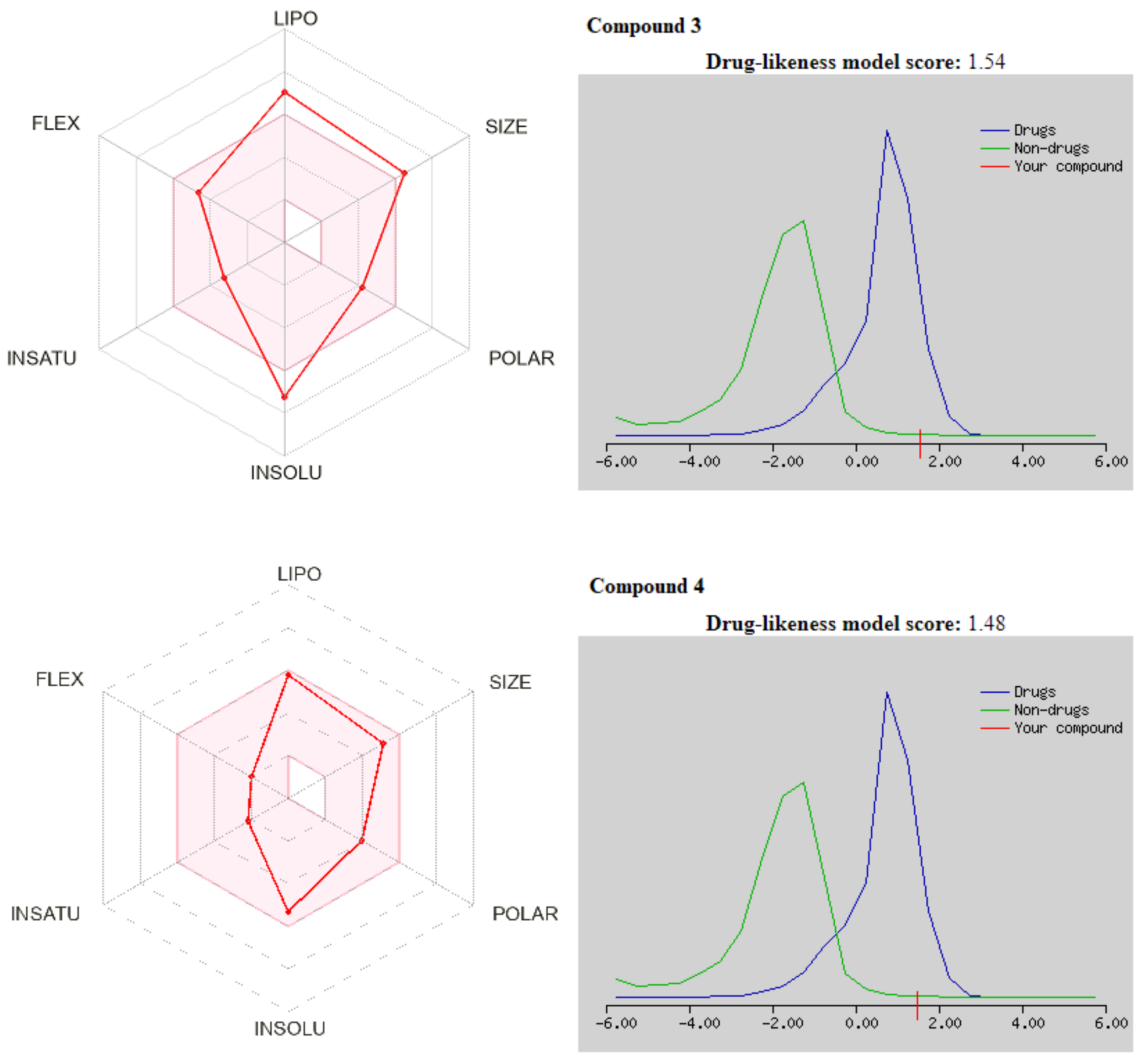
Drug-likeness model and bioavailability radar of the compounds **3** and **4**. The pink area represents the optimal range for each property for oral bioavailability, (Lipophilicity (LIPO): XLOGP3 between −0.7 and +5.0, Molecular weight (SIZE): MW between 150 and 500 g/mol, Polarity (POLAR) TPSA between 20 and 130 Å^2^, Solubility (INSOLU): log S not higher than 6, Saturation (INSATU): fraction of carbons in the sp^3^ hybridization not less than 0.25 and Flexibility (FLEX): no more than 9 rotatable bonds.

All tested compounds were evaluated for their drug-likeness and bioavailability scoresand the results are presented in Table 4. According to the prediction, the bioavailability score for most of the compounds was about 0.55, except for compounds **3**, **9**, **10** and **12** with 0.17 values. Despite these compounds exhibiting two violations of Lipinski’s rule of five, they have excellent drug-likeness scores ranging from 0.74 to 1.54. Thus, it can be concluded that they have good oral bioavailability and drug-likeness profile (Figure 7).

## 3. Materials and Methods

### 3.1. Chemistry—General Information

All commercially available reagents, isonicotinic acid hydrazide, *m*-bromobenzoic acid hydrazide, *m*-nitrobenzoic acid hydrazide and androsta-1,4-diene-3,17-dione were of analytical grade and used without further purification (all from Sigma Aldrich, Schnelldorf, Germany).

^1^H NMR and ^13^C NMR spectra were recorded using DMSO-d_6_ or CDCl_3_ as a solvent at 22 °C with a Bruker AC 400 instrument. IR spectra were recorded using a JASCO FT/IR-4600 spectrometer. Mass spectra were obtained on an HPLC-APCIMS (positive mode)-Agilent 1100 Series with an Inertsil PREP-ODS column (6.0 × 250 mm) and elution of steroids by H_2_O–MeCN (20:80). The melting points were recorded on a Gallenkamp apparatus and are uncorrected. The IR, ^1^HNMR and ^13^C NMR spectra can be found in Supplementary Materials.

3β-Phenylacetoxy-5α-androstan-17-one isonicotinoylhydrazone (**3**)

A mixture of 3β-phenylacetoxy-5α-androstan-17-one (1 g, 2.44 mmol), isonicotinic acid hydrazide (0.39 g, 2.92 mmol) and acetic acid (1 mL) in ethanol (20 mL) was boiled for 2 h and cooled to room temperature. The resulting precipitate was filtered off, washed with water and crystallized from ethanol. Yield 75%, m.p. 180–182 °C. IR(KBr): 3169, 1729, 1639, 1598, 1548, 1494, 1452, 1132, 1010, 928, 841 cm^−1^. ^1^H NMR (400 MHz, CDCl_3_, δ, ppm, J/Hz): δ, 0.89 and 1.00 (6H, s, 18-CH_3_, 19-CH_3_), 3.62 (2H, s, CH_2_C_6_H_5_), 4.75 (1H, m, H-3), 7.28–7.37 (5H, m, C_6_H_5_), 7.65–7.80 (2H, H-Pr), 8.50 (1H, s, NHCO), 8.75–8.79 (2H, dd, J = 5.8, J = 2.2, H-Pr). ^13^C NMR spectrum (100 MHz, CDCl_3_, δ, ppm): 12.3, 16.9, 20.7, 23.5, 25.3, 26.2, 27, 4, 28.3, 31.4, 33.9, 34.9, 35.7, 36.7, 41.8, 44.8, 44.9, 45.5, 53.4, 54.5, 74.0, 121.0, 124.0, 126.9, 128.5, 129.2, 134.4, 140.7, 141.1, 149.6, 150.7, 167.6, 168.1, 171.2, 174.0.

3α-Hydroxy-5α-androstan-17-one *m*-bromobenzoylhydrazone (**12**)

A mixture of 3α-hydroxy-5α-androstan-17-one (0.05 g, 0.14 mmol), m-bromobenzoic acid hydrazide (0.04 g, 0.2 mmol) and 0.1 mL acetic acid was refluxed in ethanol for 5 h. The resulting precipitate was filtered off, washed with water and crystallized from ethanol. Yield 80%, m.p. 266–268 °C. IR(KBr): 3465, 3360, 1675, 1643, 1609, 1565, 1518, 1363, 1259, 1003, 930, 890, 606 cm^−1^. ^1^H NMR (400 MHz, DMSO-d_6_, δ, ppm, J/Hz): δ, 0.78 (3H, s, 18-CH_3_), 0.86(3H, s, 19-CH_3_), 2.36–2.60 (2H, m, H-16), 4.47 (1H, s, H-3), 7.44 (1H, m, H-Ar), 7.75 (2H, m, H-Ar), 7.95 (1H, s, H-Ar), 10.34 (1H, s, NHCO). ^13^C NMR spectrum (100 MHz, DMSO-d_6_, δ, ppm J/Hz): 12.0, 16.7, 20.2, 22.7, 26.7, 28.1, 30.8, 31.2, 34.5, 35.0, 36.4, 37.9, 44.3, 44.5, 52.7, 53.9, 69.1, 121.4, 126.6, 130.2, 133.8, 136.4, 150.4, 161.8, 175.1.

### 3.2. In Vitro Evaluation of Antimicrobial Activity

The following Gram-negative bacteria: *Escherichia coli* (ATCC 35210), *Salmonella* Typhimurium (ATCC 13311), *Pseudomonas aeruginosa* (ATCC 27853) as well as Gram-positive bacteria*: Listeria monocytogenes* (NCTC 7973), *Bacillus cereus* (clinical isolate) and *Staphylococcus aureus* (ATCC 6538) were used. Resistant strains used were Methicillin resistant *S. aureus* (IBRS MRSA 011), resistant *E. coli* (IBRS E003) and resistant *P. aeruginosa* (IBRS P001) obtained as described in the previous paper [31]. The organisms were obtained from the Mycological Laboratory, Department of Plant Physiology, Institute for Biological Research “Siniša Stankovic”, National Institute of Republic of Serbia, Belgrade, Serbia.

The antifungal activity of all investigated samples was tested on strains obtained from the Mycological Laboratory, Department of Plant Physiology, Institute for Biological Research “Sinisa Stankovic”, National Institute of Republic of Serbia, Belgrade, Serbia. The following fungi *Aspergillus fumigatus* (ATCC 1022), *Aspergillus niger* (ATCC 6275), *Trichoderma viride* (IAM 5061), *Penicillium funiculosum* (ATCC 36839), *P. verrucosum var. cyclopium* (food isolates) and *Candida albicans* (ATCC 10231) were tested. The detailed explanation is given in our previous papers [32,33].

Commercial antibiotics, ampicillin and streptomycin, and fungicides, bifonazole and ketoconazole, were used as positive controls. EtOH 30% was used as a negative control. All experiments were performed in duplicate and repeated three times.

The minimum inhibitory (MIC) and minimum bactericidal/fungicidal (MBC/MFC) concentrations were determined by the modified microdilution method, as previously reported [34,35].

#### Inhibition of Biofilm Formation

The potential of compounds to inhibit biofilm formation was investigated as previously described, with some modifications [36] *C. albicans* ATCC 10231 was incubated in 96-well microtiter plates with an adhesive bottom (Sarstedt, Germany), with MIC and sub-MIC concentrations of tested compounds/referent drug in YPD medium at 37 °C for 24 h. Afterwards, wells were washed thrice with sterile PBS (Phosphate buffered saline, pH 7.4) and biofilms were fixed with methanol for 20 min. Then, methanol was removed and stained with 0.1% crystal violet (Bio-Merieux, France) for 30 min. The plate was slowly washed, air dried and 96% ethanol (Zorka, Serbia) was added to dissolve bounded crystal violet. The absorbance (620 nm) was read on a Multiskan™ FC Microplate Photometer, Thermo Scientific™. Lastly, the percentage of inhibition of biofilm formation was calculated by the formula:

Percentage of inhibition = ((A_620(control)_ − A_620sample_)/A_620control_)) × 100

### 3.3. Docking

AutoDock 4.2^®^ software was used for the in silico studies and a detailed procedure is reported in our previous paper [37].

### 3.4. Drug-Likeness

Drug-likeness [38] scores of compounds were predicted using the Molsoft software and SwissADME program (http://swissadme.ch, accessed on 19 October 2022) via the ChemAxon’s Marvin JS structure drawing tool [39].

## 4. Conclusions

This work presents the synthesis of two new steroid derivatives and the study of antibacterial and antifungal activities together with previously synthesized compounds against a panel of bacterial and fungal pathogens of twelve steroid derivatives, two of which are new. The antibacterial activity of tested compounds was low to moderate with minimal inhibitory concentration being 0.37–1.5 mg/mL and minimal bactericidal being 1.5–3.0 mg/mL, except against *B. cereus* which was good. The antibacterial activity against resistant strains MRSA, *E. coli* and *P. aeruginosa* was superior against MRSA than ampicillin, which did not show bacteriostatic or bactericidal activity, while against the two other strains, it did not show bactericidal activity. All compounds exhibited moderate to good antifungal potency with MIC and MFC in the range of 0.37–3.00 mg/mL and 0.50–6.00 mg/mL, respectively. Compound **7** demonstrated the best activity among all tested with MIC/MFC of 0.37/0/75 mg/mL, respectively. The most sensitive fungal to compounds tested was *T. viride*, while *C. albicans* was the most resistant one. Despite this, almost all compounds except for **11** and **12** were more potent than ketoconazole against *C. albicans,* the deathiest fungal. Antibiofilm activity assessed for the two most potent compounds **1** and **8** in concentrations of MIC, 0.5 MIC and 0.25 MIC revealed that it was lower (33 and 15%, respectively, in concentration of MIC) than that of ketoconazole. According to docking results, it seems that the inhibition of CYP51 reductase is responsible for the antifungal activity of the compounds. All compounds showed good drug-likeness scoresin the range of 0.71–1.54. Three compounds showed two violations to the Lipinski rule.

## Figures and Tables

**Table 1:** Antibacterial activity of compounds **1**–**12** (MIC/MBC in mg/mL).

Compounds		*S.a.*	*MRSA*	*B.c*	*L.m.*	*E. coli*	*Rez E. coli*	*P.a.*	*Rez P.a.*	*S.Thy*
**1**	**MIC**	1.50	1.50	0.37	0.75	1.00	4.50	1.50	1.00	0.75
	**MBC**	3.00	3.00	0.75	1.50	3.00	6.00	3.00	1.50	1.50
**2**	**MIC**	3.00	4.50	0.75	3.00	3.00	6.00	6.00	6.00	6.00
	**MBC**	6.00	6.00	1.50	6.00	6.00	9.00	9.00	9.00	9.00
**3**	**MIC**	1.00	0.75	0.37	1.00	1.00	1.50	1.50	0.75	1.00
	**MBC**	1.50	1.50	0.75	1.50	1.50	3.00	3.00	1.50	1.50
**4**	**MIC**	0.75	3.00	0.37	0.75	1.50	1.50	0.75	0.75	0.75
	**MBC**	1.50	6.00	0.75	1.50	3.00	3.00	1.50	1.50	1.50
**5**	**MIC**	0.75	1.50	0.37	0.75	1.50	1.50	0.75	1.00	0.75
	**MBC**	1.50	3.00	0.75	1.50	3.00	3.00	1.50	1.50	1.50
**6**	**MIC**	0.75	1.50	0.37	1.50	1.50	0.75	3.00	1.50	1.50
	**MBC**	1.50	3.00	0.75	3.00	3.00	1.50	6.00	3.00	3.00
**7**	**MIC**	0.75	1.50	0.50	1.50	1.50	0.75	3.00	1.50	1.50
	**MBC**	1.50	3.00	0.75	3.00	3.00	1.50	6.00	3.00	3.00
**8**	**MIC**	1.50	1.50	0.37	1.50	1.50	1.50	3.00	1.50	1.50
	**MBC**	3.00	3.00	0.75	3.00	3.00	3.00	6.00	3.00	3.00
**9**	**MIC**	1.50	3.00	1.50	0.75	3.00	6.00	1.50	3.00	3.00
	**MBC**	3.00	6.00	3.00	1.50	6.00	9.00	3.00	6.00	6.00
**10**	**MIC**	1.50	3.00	1.50	0.75	3.00	6.00	1.50	1.50	0.75
	**MBC**	3.00	6.00	3.00	1.50	6.00	9.00	3.00	3.00	1.50
**11**	**MIC**	0.75	1.50	0.75	0.75	0.75	1.50	0.75	0.75	0.75
	**MBC**	1.50	3.00	1.50	1.50	1.50	3.00	1.50	1.50	1.50
**12**	**MIC**	0.75	1.50	0.75	0.75	0.75	1.50	0.75	0.75	0.75
	**MBC**	1.50	3.00	1.50	1.50	1.50	3.00	1.50	1.50	1.50
**Ampicillin**	**MIC**	0.10	-	0.10	0.15	0.15	0.20	0.30	0.20	0.10
	**MBC**	0.15	-	0.15	0.30	0.20	-	0.50	-	0.20
**Streptomycin**	**MIC**	0.10	0.10	0.025	0.15	0.10	0.05	0.10	0.10	0.10
	**MBC**	0.20	-	0.050	0.30	0.20	0.10	0.20	0.20	0.20

**Table 2:** Antifungal activity of compounds **1**–**12** (MIC/MBC in mg/mL).

Compounds		*A.fu*	*A.n.*	*T.v.*	*P.f.*	*P.v.c.*	*C.a.*
**1**	**MIC**	0.75	0.75	0.37	0.75	0.37	0.37
	**MFC**	1.50	1.50	0.75	1.50	0.75	0.75
**2**	**MIC**	1.50	1.50	1.50	1.50	3.00	0.37
	**MFC**	3.00	3.00	3.00	3.00	6.00	0.75
**3**	**MIC**	0.37	0.37	0.37	0.37	0.37	0.75
	**MFC**	0.75	0.75	0.75	0.75	0.75	1.50
**4**	**MIC**	1.50	3.00	3.00	3.00	3.00	0.75
	**MFC**	3.00	6.00	6.00	6.00	6.00	1.50
**5**	**MIC**	1.50	1.50	1.50	1.50	0.75	0.75
	**MFC**	3.00	3.00	3.00	3.00	1.50	1.50
**6**	**MIC**	1.50	1.50	1.50	0.75	0.75	0.75
	**MFC**	3.00	3.00	3.00	1.50	1.50	1.50
**7**	**MIC**	0.37	0.37	0.37	0.37	0.37	0.37
	**MFC**	0.75	0.75	0.75	0.75	0.75	0.75
**8**	**MIC**	0.37	0.37	0.37	0.37	0.37	0.37
	**MFC**	0.75	0.75	0.75	0.75	0.75	0.75
**9**	**MIC**	0.37	0.75	0.37	0.75	0.75	0.37
	**MFC**	0.75	1.50	0.75	1.50	1.50	0.75
**10**	**MIC**	0.37	1.50	0.75	1.50	3.00	0.37
	**MFC**	0.50	3.00	1.50	3.00	6.00	0.75
**11**	**MIC**	0.75	1.50	0.75	0.75	1.50	1.50
	**MFC**	1.50	3.00	1.50	1.50	3.00	3.00
**12**	**MIC**	0.37	0.37	0.37	0.75	1.50	1.50
	**MFC**	0.50	0.50	0.75	1.50	3.00	3.00
**Ketoconazole**	**MIC**	0.20	0.20	1.00	0.20	0.20	1.00
	**MFC**	0.50	0.50	1.50	0.50	0.30	2.00
**Bifonazole**	**MIC**	0.15	0.15	0.15	0.20	0.10	0.20
	**MFC**	0.20	0.20	0.20	0.25	0.20	0.30

**Table 3:** Molecular docking free binding energies (kcal/mol) to antifungal targets.

	Est. Binding Energy (kcal/mol)		Residues Involved in H-bond Formation	Residues Involved in Hydrophobic Interactions	Residues Involved in Aromatic Interactions	Interactions with HEM601
Comp.	CYP51 of *C. albicans* 5V5Z	DNA TopoIV 1S16				
**1**	−4.11	−8.52	Tyr132	Tyr118, Thr122, Thr311, Phe380, Met508, Hem601	Hem601	Hydrophobic, aromatic
**2**	−3.31	−6.38	-	Tyr118, Thr311, Leu376, Hem601	-	Hydrophobic
**3**	−3.58	−9.04	Met508	Tyr118, Thr122, Phe228, Phe233, Thr311, Leu376, Phe380, Met508, Hem601	Hem601	Hydrophobic, aromatic
**4**	−1.77	−6.01	-	Tyr118, Leu376, Met508, Hem601	-	Hydrophobic
**5**	−2.47	−6.80	-	Tyr118, Tyr122, Leu376, Met508, Hem601	Tyr118	Hydrophobic
**6**	−4.20	−7.32	-	Tyr118, Tyr122, Thr311, Leu37, Hem601	Tyr118	Hydrophobic
**7**	−3.18	−9.86	Tyr132	Tyr118, Leu121, Thr311, Met508, Hem601	Hem601	Hydrophobic, aromatic
**8**	−2.54	−10.23	Tyr64	Tyr118, Thr122, Ile131, Tyr132, Leu376, Met508, Hem601	-	Hydrophobic, Fe binding
**9**	−3.61	−8.48	Tyr132	Tyr118, Leu121, Thr122, Thr311, Met508, Hem601	Hem601	Hydrophobic, aromatic
**10**	−1.38	−7.11	-	Tyr118, Thr311, Met508, Hem601	Tyr118	Hydrophobic
**11**	−2.47	−7.55	Tyr118	Tyr118, Leu121, Met508, Hem601	-	Hydrophobic
**12**	−1.28	−8.02	Tyr118	Tyr118, Tyr122, Thr311, Met508, Hem601	Hem601	Hydrophobic, aromatic
**ketoconazole**	-	−8.93	Tyr64	Tyr118, Ile131, Tyr132, Leu300, Ile304, Leu376, Met508, Hem601	Hem601	Hydrophobic, aromatic

**Table 4:** Drug-likeness predictions of tested compounds.

No	MW	Number of HBA ^a^	Number of HBD ^b^	Log *P*_o/w_ (WLOGP) ^c^	Log S ^d^	TPSA ^e^	Lipinski	Bioavailability Score	Drug-Likeness Model Score
**1**	522.64	6	2	5.09	Moderately soluble	108.70	0	0.55	1.09
**2**	448.60	6	1	6.28	Moderately soluble	104.10	0	0.55	1.30
**3**	541.72	5	1	6.75	Moderately soluble	80.65	2 violations: MW > 500, MLOGP > 4.15	0.17	1.54
**4**	423.59	4	2	3.75	Moderately soluble	74.58	0	0.55	1.48
**5**	405.64	2	3	4.61	Moderately soluble	102.73	0	0.55	0.71
**6**	375.57	3	2	3.44	Moderately soluble	102.73	0	0.55	0.74
**7**	407.55	4	2	4.49	Moderately soluble	74.78	0	0.55	1.29
**8**	430.66	2	4	3.10	Soluble	165.00	0	0.55	0.78
**9**	509.64	6	1	6.05	Poorly soluble	113.58	2 violations: MW > 500, MLOGP > 4.15	0.17	0.98
**10**	543.54	4	1	6.90	Poorly soluble	67.76	2 violations: MW > 500, MLOGP > 4.15	0.17	1.18
**11**	467.60	5	2	5.47	Moderately soluble	107.51	0	0.55	0.85
**12**	501.50	3	2	6.33	Moderately soluble	61.69	2 violations: MW > 500, MLOGP > 4.15	0.17	1.07

^a^ number of hydrogen bond acceptors; ^b^ number of hydrogen bond donors; ^c^ lipophilicity; ^d^ Water solubility (SILICOS-IT [S = Soluble]); ^e^ topological polar surface area (Å^2^).

## Data Availability

Not applicable.

## Supplementary Materials

The following supporting information can be downloaded at: https://www.mdpi.com/article/10.3390/molecules28031167/s1; Figure S1: ^13^C NMR spectrum of compound **1** in DMSO-d6; Figure S2: ^1^H NMR spectrum of 3β-azido-5α-androstan-17-one isonicotinoylhydrazone (compound **2**) in CDCl_3_; Figure S3: ^13^C NMR spectrum of 3β-azido-5α-androstan-17-one isonicotinoylhydrazone (compound **2**) in CDCl_3_; Figure S4: IR spectrum of 3β -phenylacetoxy-5α-androstan-17-one isonicotinoylhydrazone (compound **3**) in KBr; Figure S5: ^1^H NMR spectrum of 3β-phenylacetoxy-5α-androstan-17-one isonicotinoylhydrazone (compound **3**) in CDCl_3_; Figure S6: ^13^C NMR spectrum of 3β-phenylacetoxy-5α-androstan-17-one isonicotinoylhydrazone (compound **3**) in CDCl_3_; Figure S7: MS spectrum of 3β -phenylacetoxy-5α-androstan-17-one isonicotinoylhydrazone (compound **3**); Figure S8: ^13^C NMR spectrum of compound **4** in d_6_-DMSO; Figure S9: ^1^H NMR spectrum of 3α-hydroxy-5α-pregnan-20-one thiosemicarbazone (compound **5**) in d_6_-DMSO; Figure S10: ^13^C NMR spectrum of 3α-hydroxy-5α-pregnan-20-one thiosemicarbazone (compound **5**) in d_6_-DMSO; Figure S11: ^13^C NMR spectrum of compound **6** in d_6_-DMSO; Figure S12: ^1^H NMR spectrum of 3α-hydroxy-5α-androst-9(11)-en-17-one isonicotinoylhydrazone (compound **7**) in d_6_-DMSO; Figure S13: ^13^C NMR spectrum of 3α-hydroxy-5α-androst-9(11)-en-17-one isonicotinoylhydrazone (compound **7**) in d_6_-DMSO; Figure S14: ^13^C NMR spectrum of androsta-1,4-diene-3,17-dione dithiosemicarbazone (compound **8**) in d_6_-DMSO; Figure S15: ^13^C NMR spectrum of 3β-acetoxy-5α-androstan-17-one *m*-nitrobenzoylhydrazone (compound **9**) in d_6_-DMSO; Figure S16: ^13^C NMR spectrum of 3β -acetoxy-5α-androstan-17-one *m*-bromobenzoylhydrazone (compound **10**) in d_6_-DMSO; Figure S17: ^1^H NMR spectrum of 3α -hydroxy-5α-androstan-17-one *m*-nitrobenzoylhydrazone (compound **11**) in d_6_-DMSO; Figure S18: ^13^C NMR spectrum of 3α -hydroxy-5α-androstan-17-one *m*-nitrobenzoylhydrazone (compound **11**) in d_6_-DMSO; Figure S19: IR spectrum of 3α-hydroxy-5α-androstan-17-one *m*-bromobenzoylhydrazone (compound **12**) in KBr; Figure S20: ^1^H NMR spectrum of 3α-hydroxy-5α-androstan-17-one *m*-bromobenzoylhydrazone (compound **12**) in d_6_-DMSO; Figure S21: ^13^C NMR spectrum of 3α-hydroxy-5α-androstan-17-one *m*-bromobenzoylhydrazone (compound **12**) in d_6_-DMSO; Figure S22: Fragment of ^13^C NMR spectrum 3α-hydroxy-5α-androstan-17-one *m*-bromobenzoylhydrazone (compound **12**) in d_6_-DMSO; Figure S23: MS spectrum of 3α-hydroxy-5α-androstan-17-one *m*-bromobenzoylhydrazone (compound **12**).
