# Supplementary material for: Antimicrobial Activity of Some Steroidal Hydrazones
Source: Molecules. 2023 Jan 24;28(3):1167. doi: 10.3390/molecules28031167 (PMC9920613; doi:10.3390/molecules28031167)
Supplement: Supplementary file 1 [file molecules-28-01167-s001.zip › molecules-2157868-supplementary.pdf]

Supplementary materials

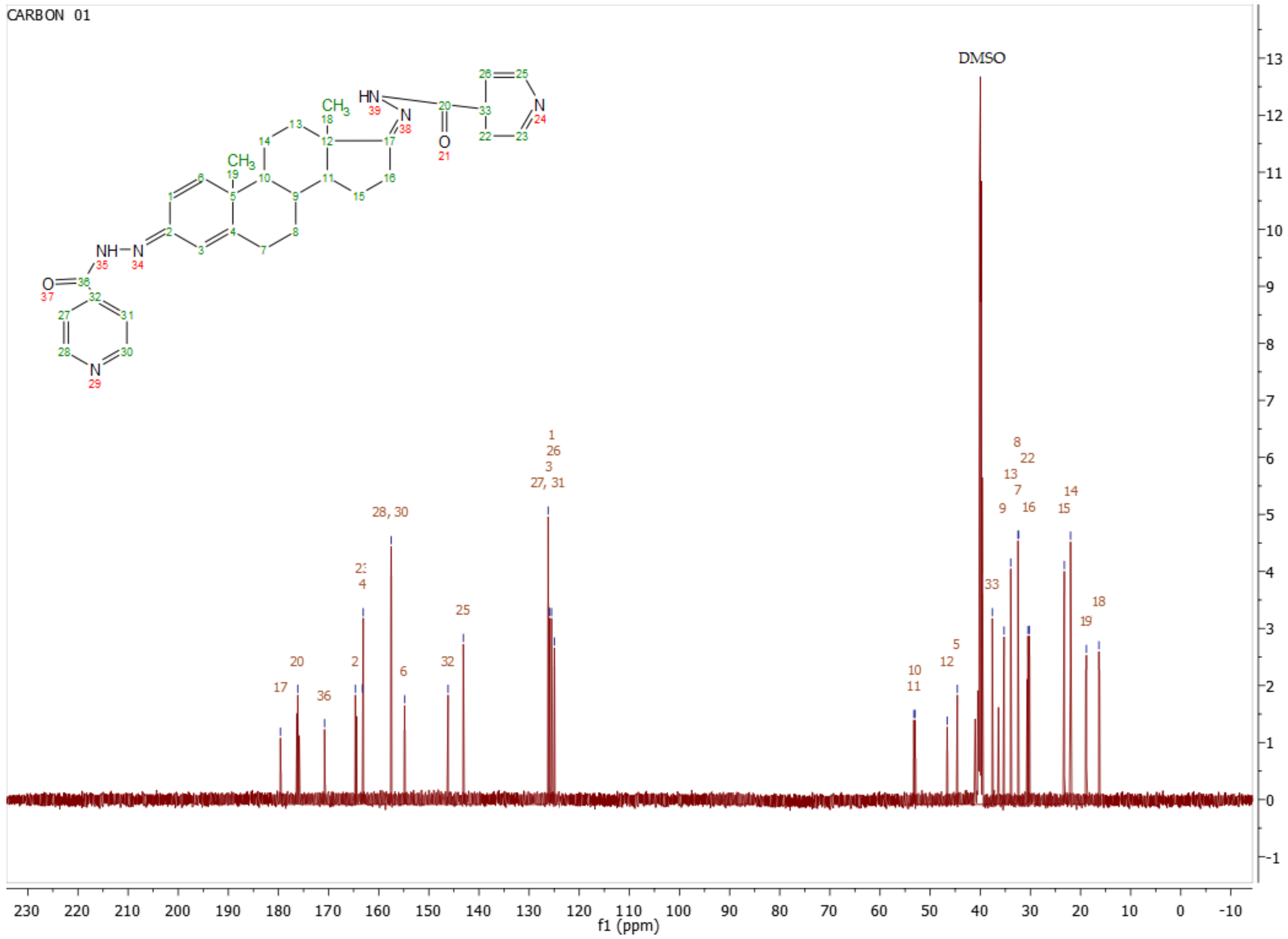

Figure S1: ^{13}C NMR spectrum of compound **1** in DMSO-d6

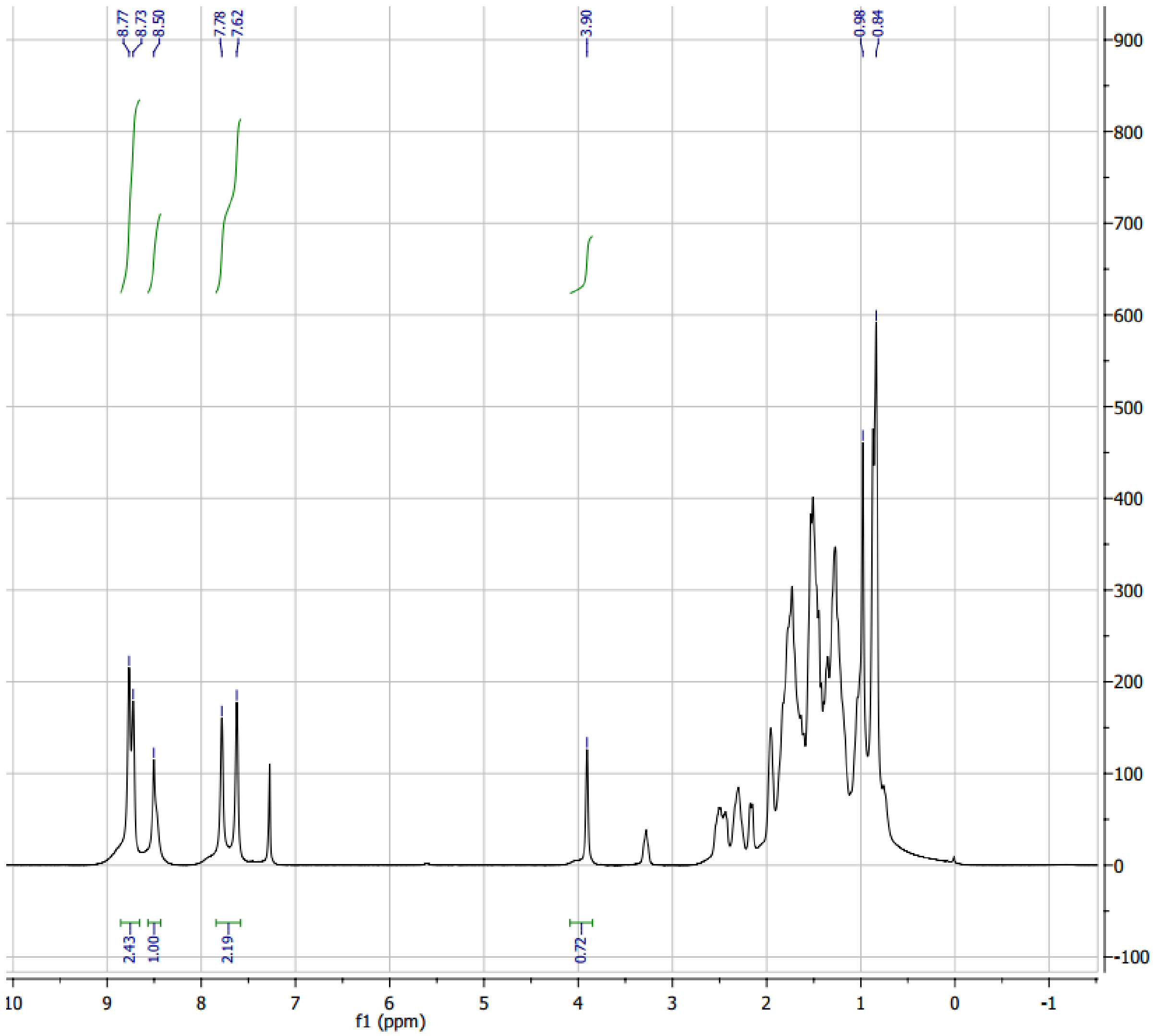

Figure S2: ^{1}H NMR spectrum of 3β-azido-5α-androstan-17-one isonicotinoylhydrazone (compound **2**) in $CDCl_3$

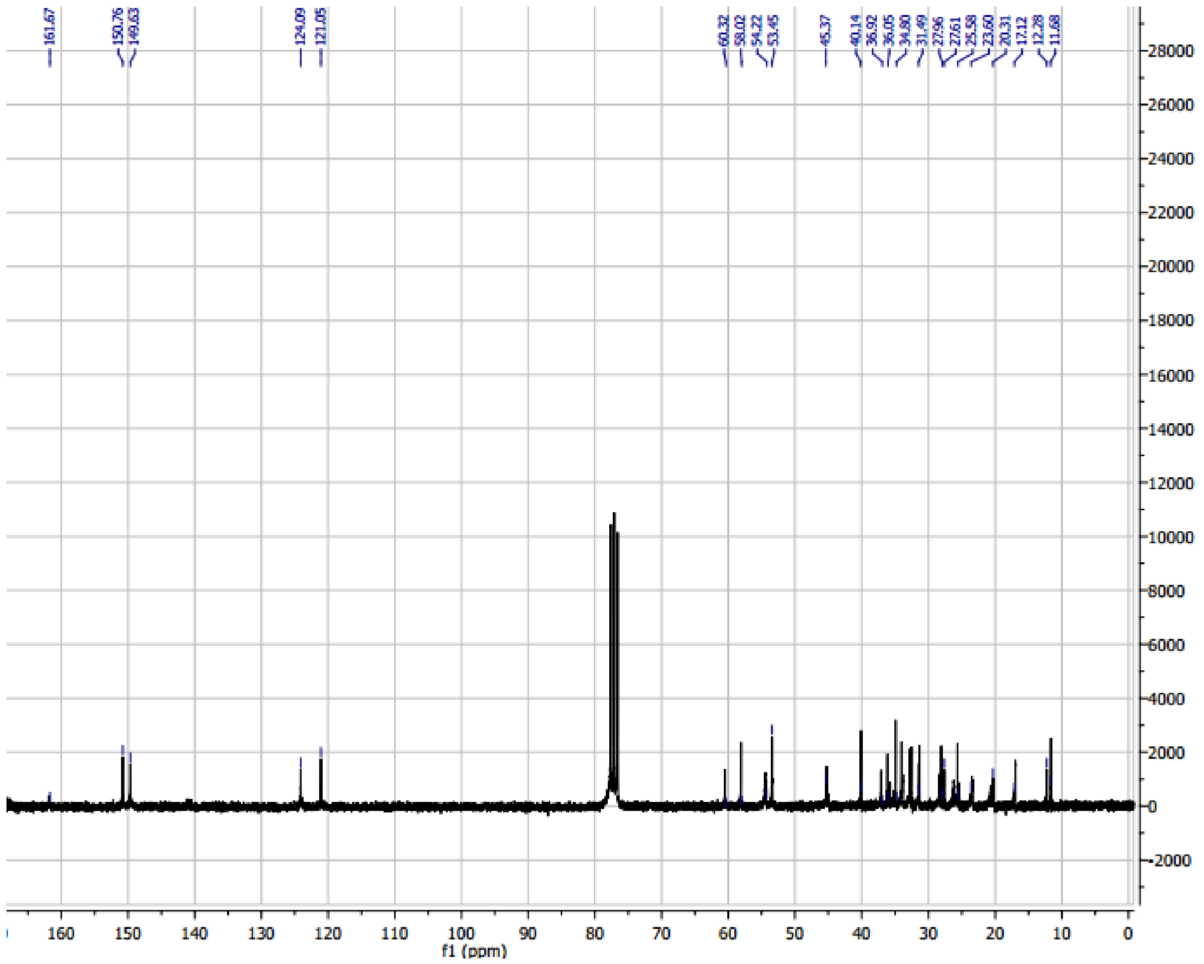

Figure S3: ^{13}C NMR spectrum of 3β-azido-5α-androstan-17-one isonicotinoylhydrazone (compound **2**) in $CDCl_3$

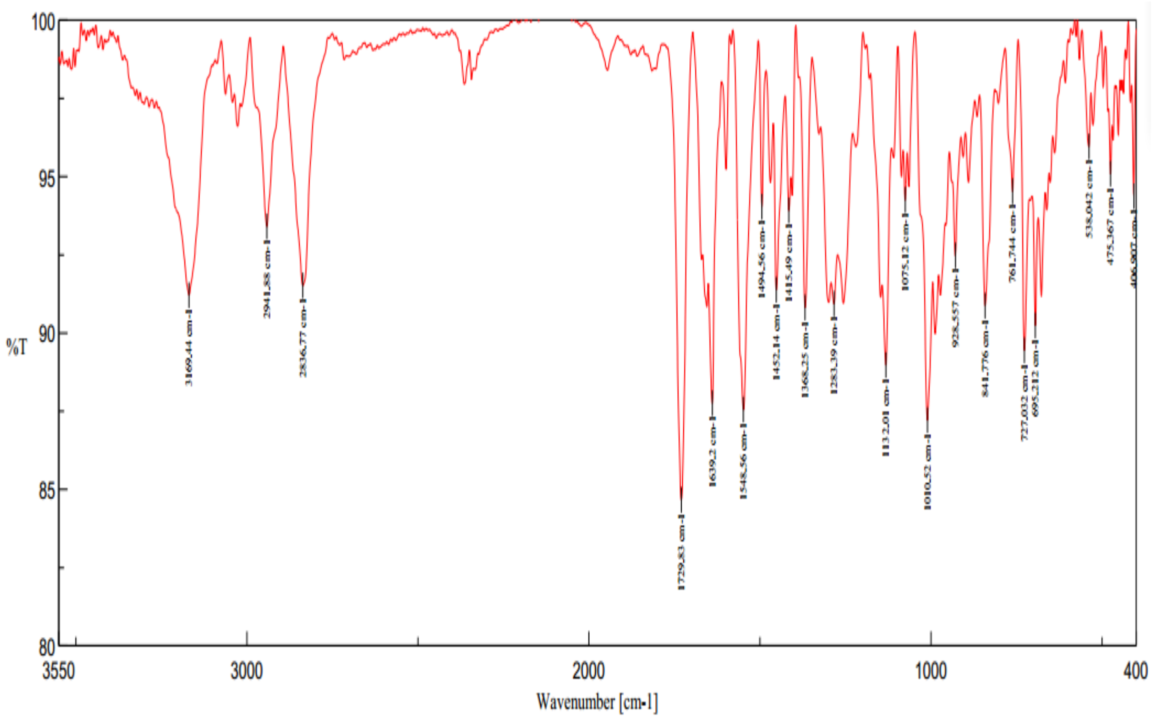

Figure S4: IR spectrum of 3β -phenylacetoxy-5α-androstan-17-one isonicotinoylhydrazone (compound **3**) in KBr

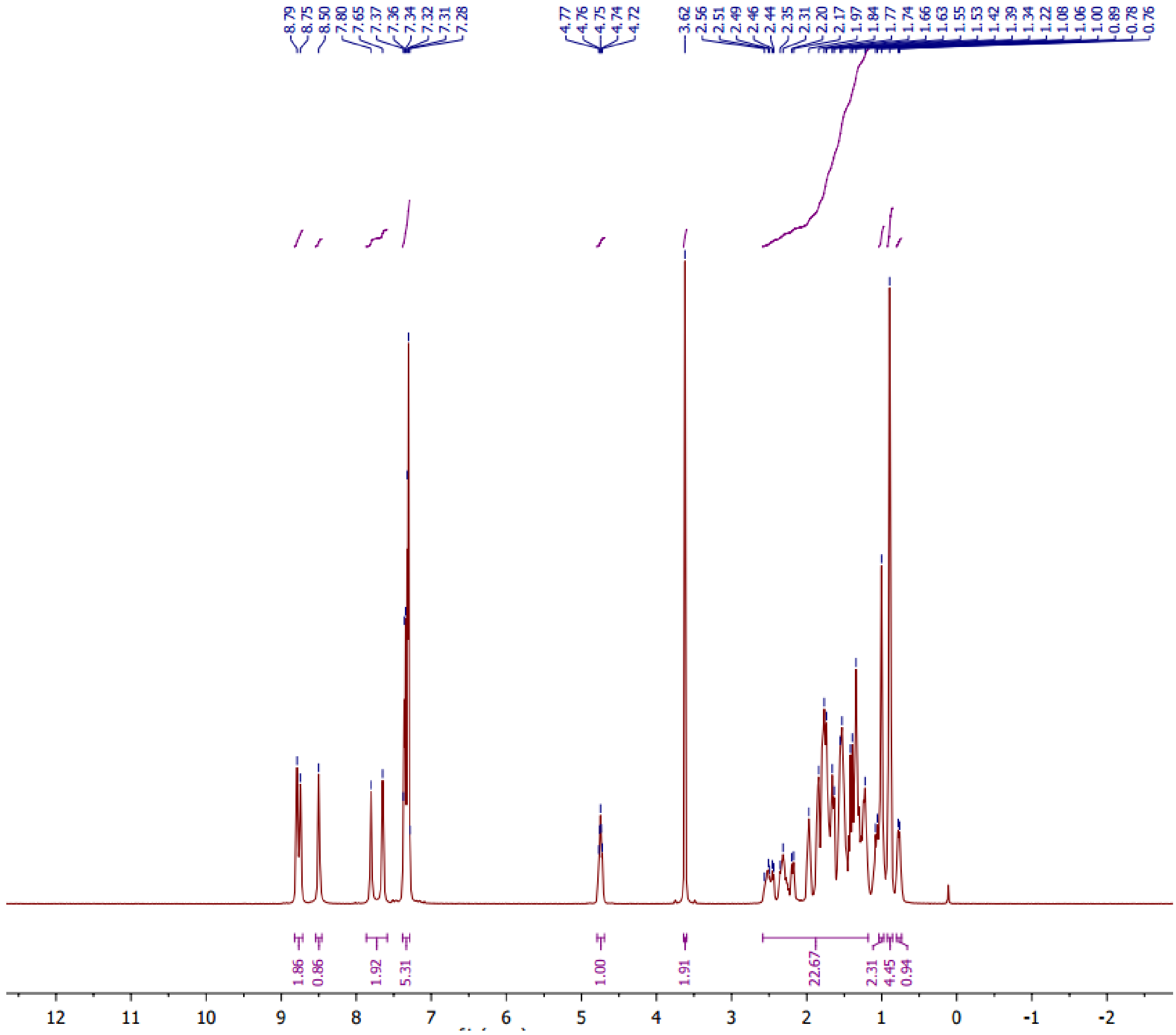

Figure S5: ^{1}H NMR spectrum of 3β-phenylacetoxy-5α-androstan-17-one isonicotinoylhydrazone (compound **3**) in $CDCl_3$

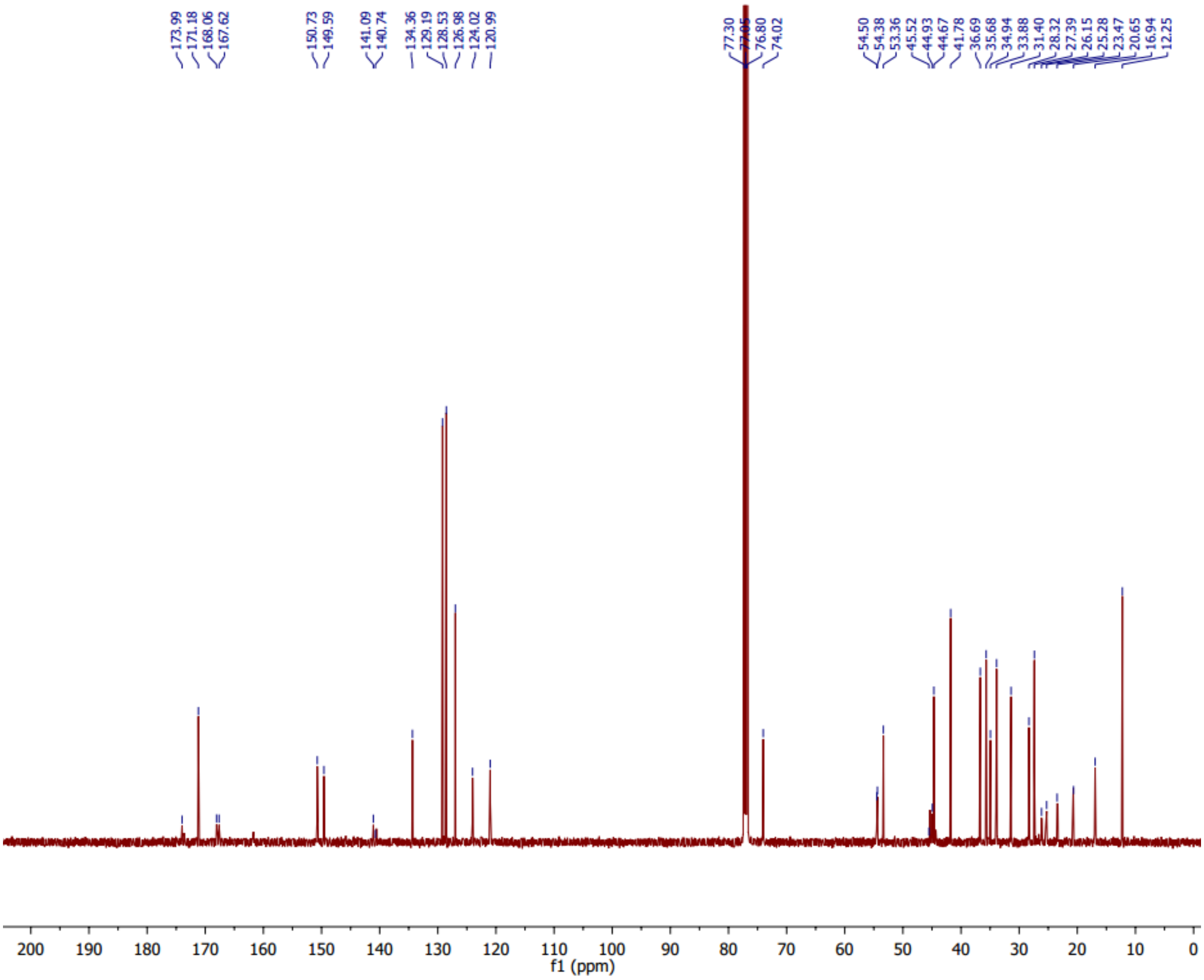

Figure S6: ^{13}C NMR spectrum of 3β-phenylacetoxy-5α-androstan-17-one isonicotinoylhydrazone (compound **3**) in $CDCl_3$

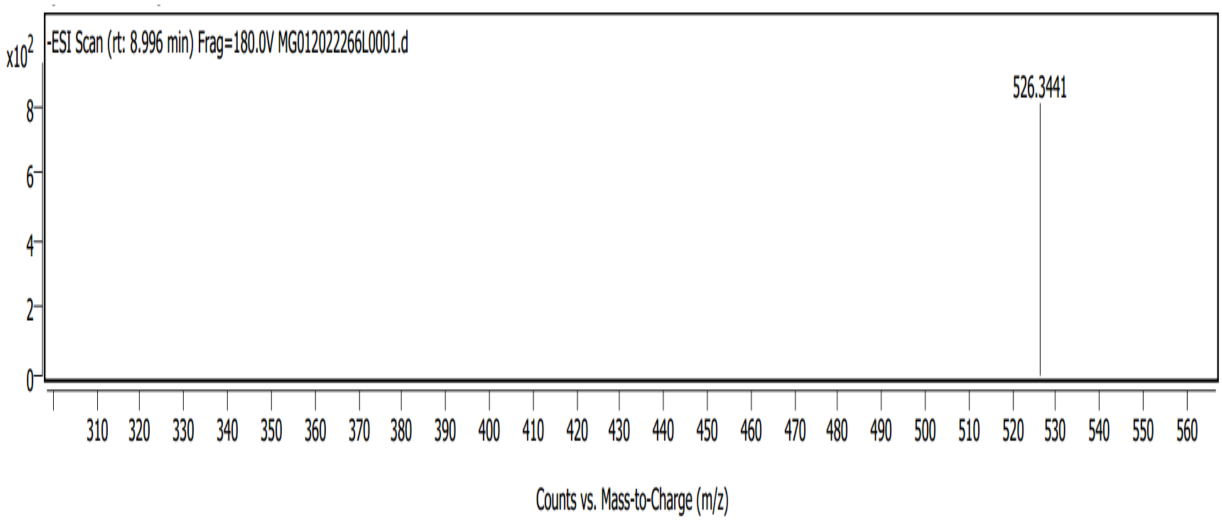

Figure S7: MS spectrum of 3β -phenylacetoxy-5α-androstan-17-one isonicotinoylhydrazone (compound **3**)

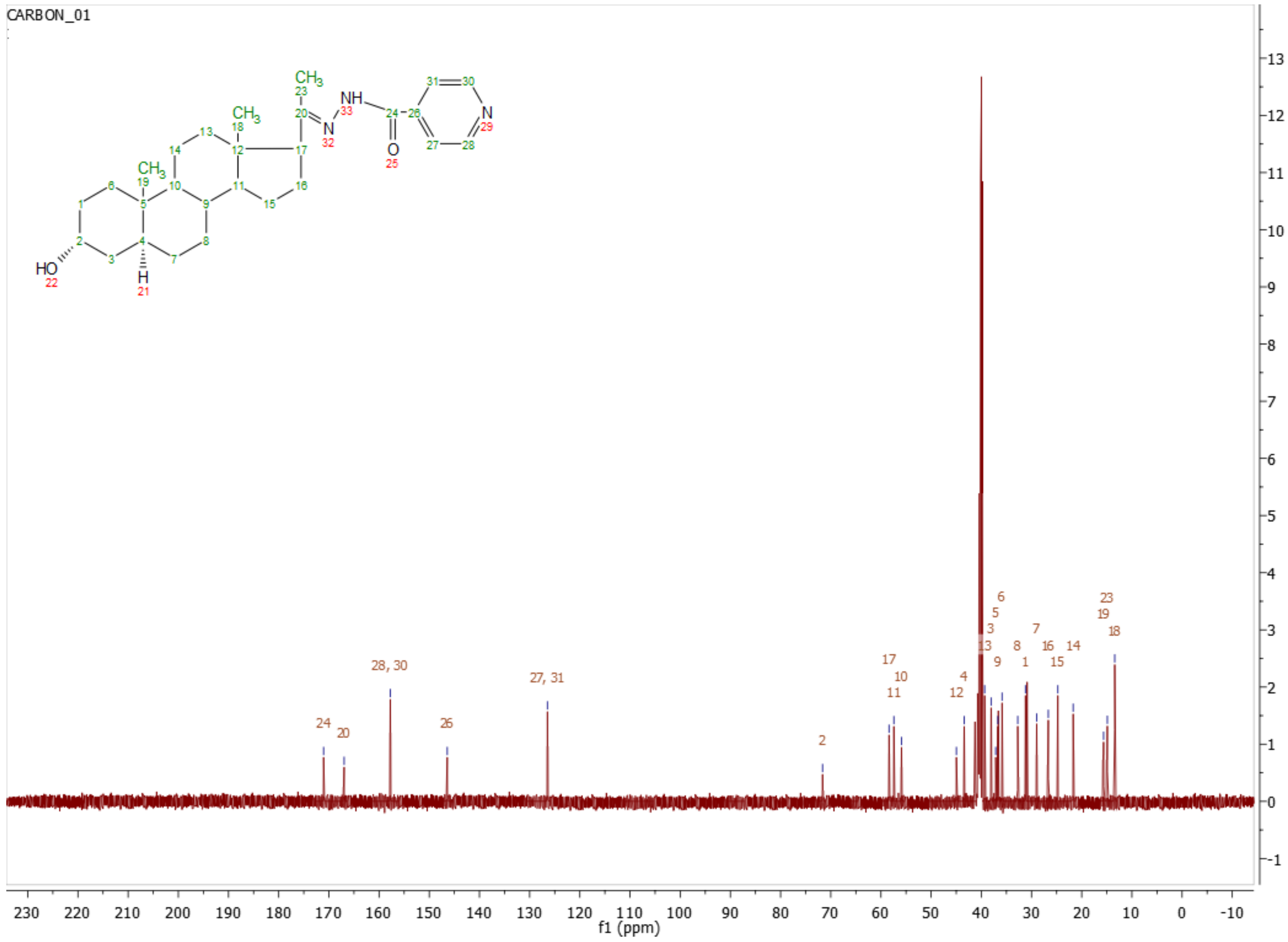

Figure S8: ^{13}C NMR spectrum of compound **4** in d_6-DMSO

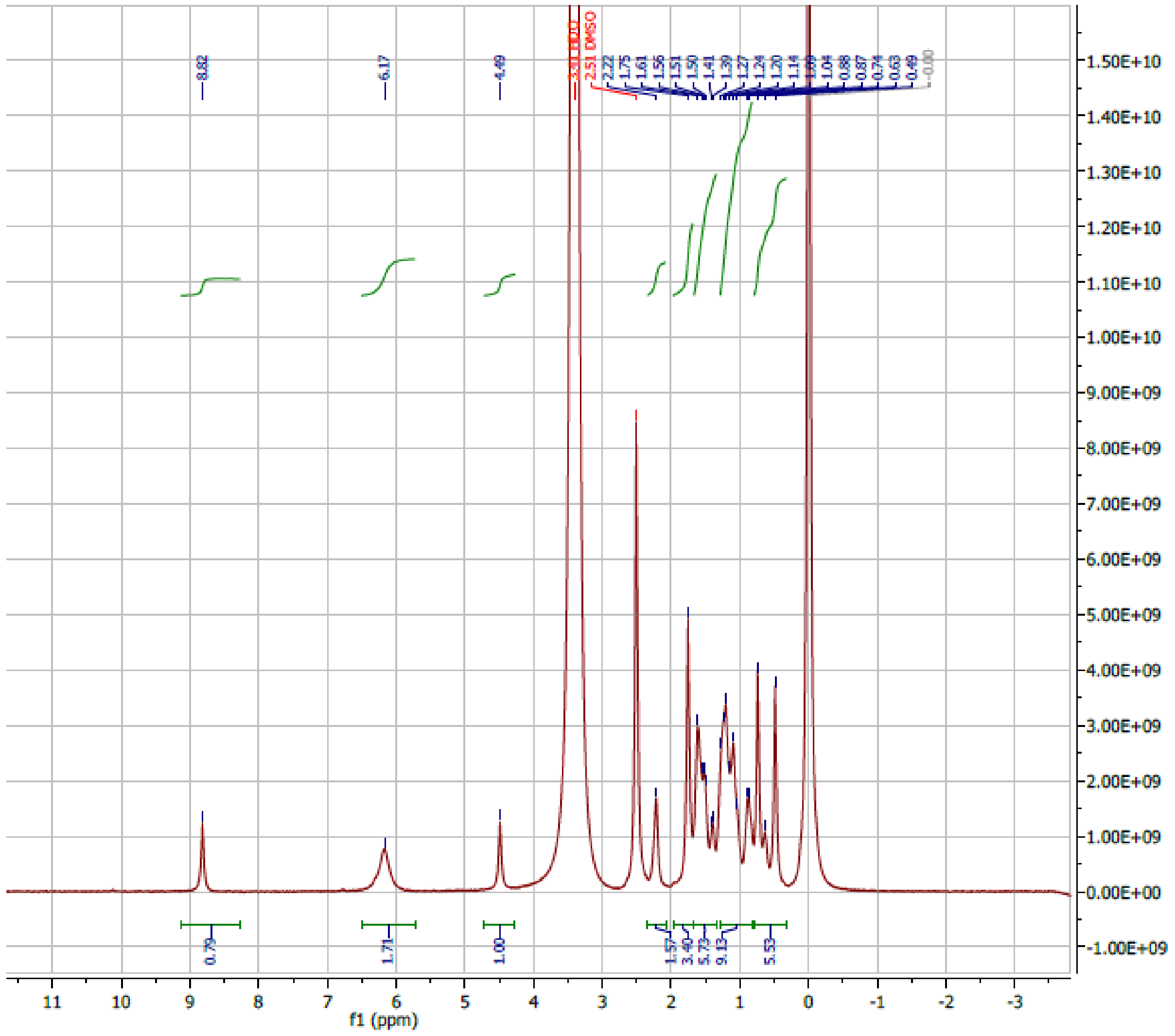

Figure S9: ^{1}H NMR spectrum of 3α-hydroxy-5α-pregnan-20-one thiosemicarbazone (compound **5**) in d_6-DMSO

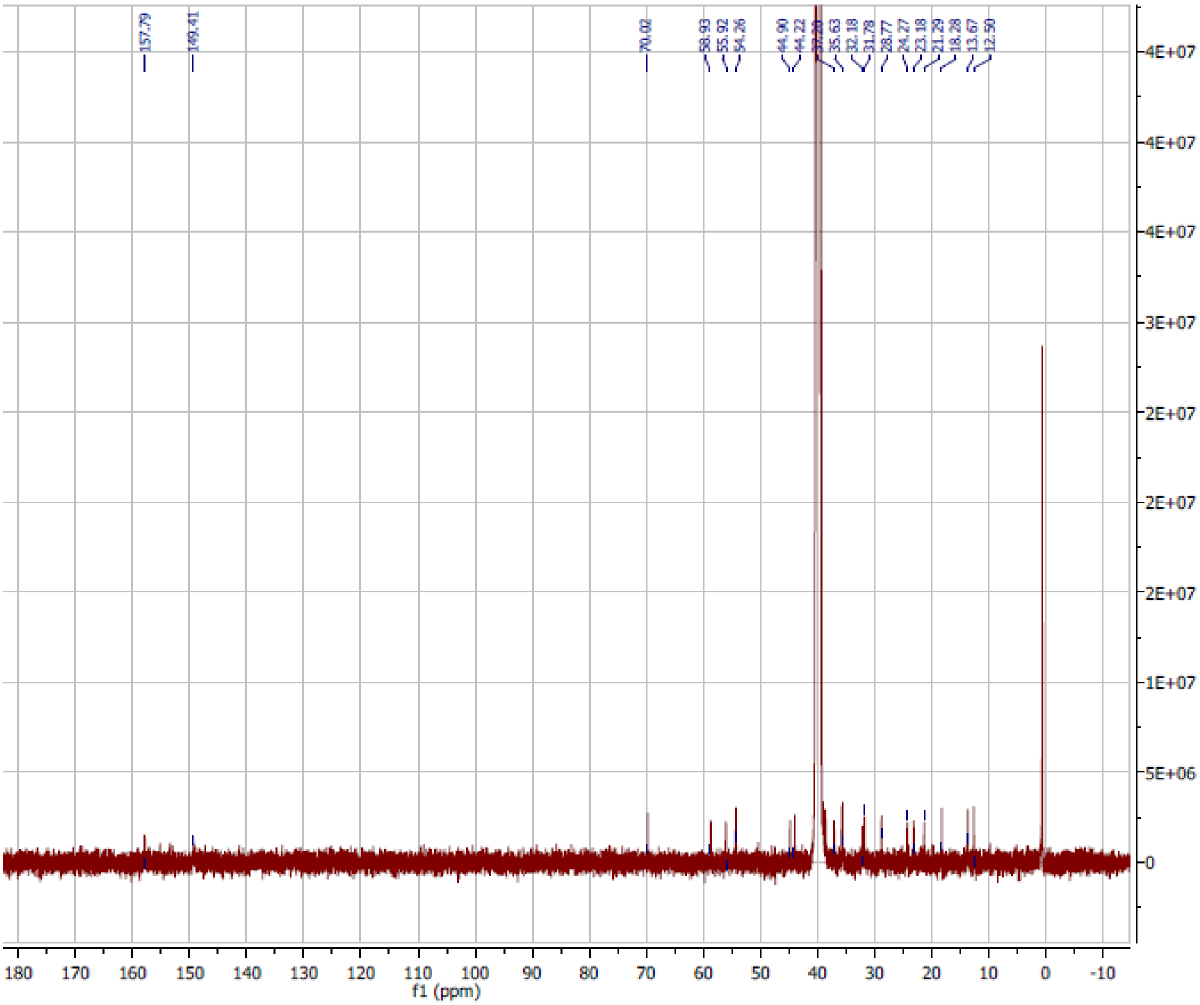

Figure 10: ^{13}C NMR spectrum of 3α-hydroxy-5α-pregnan-20-one thiosemicarbazone (compound **5**) in d_6-DMSO

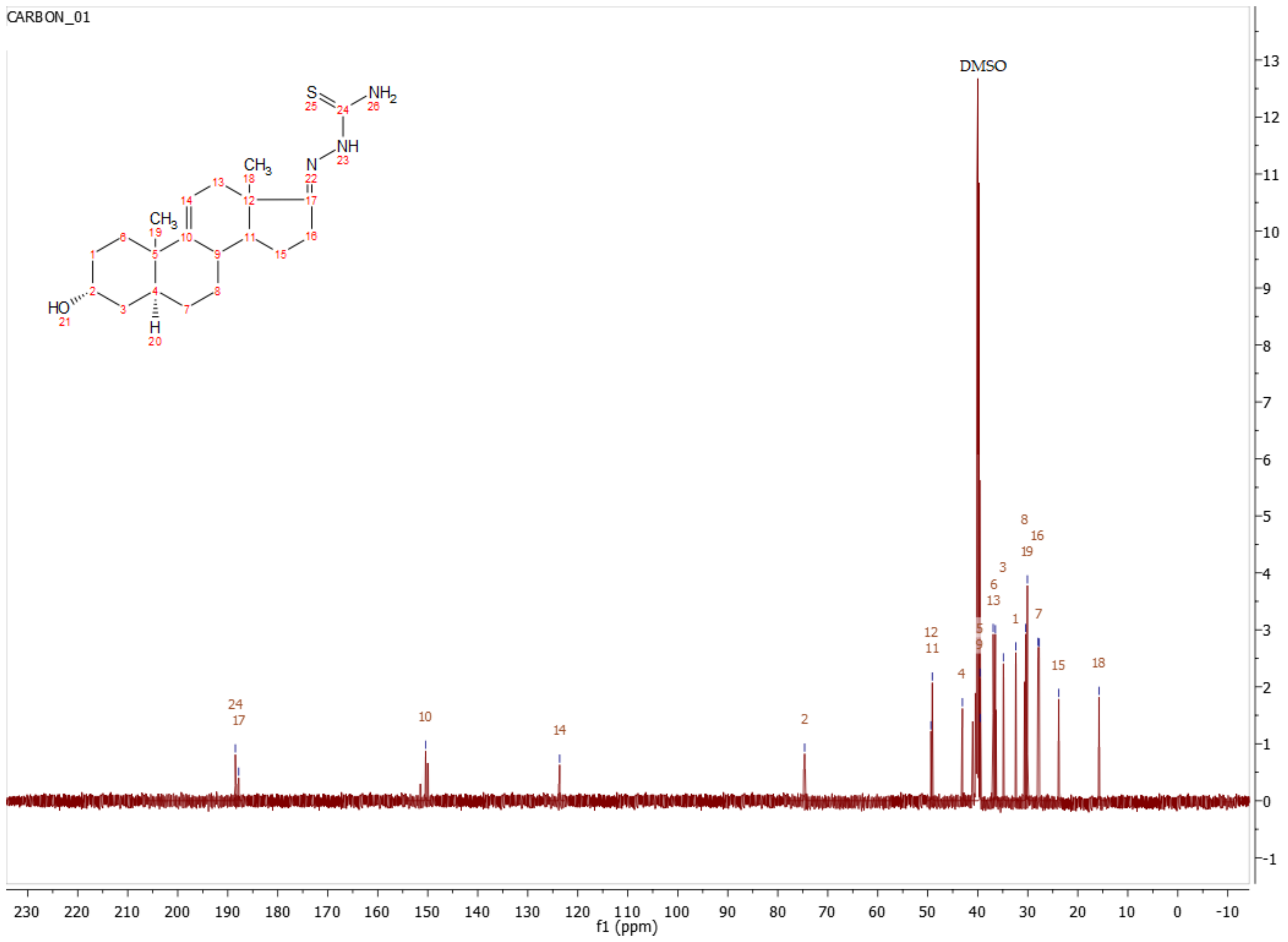

Figure S11: ^{13}C NMR spectrum of compound **6** in d_6-DMSO

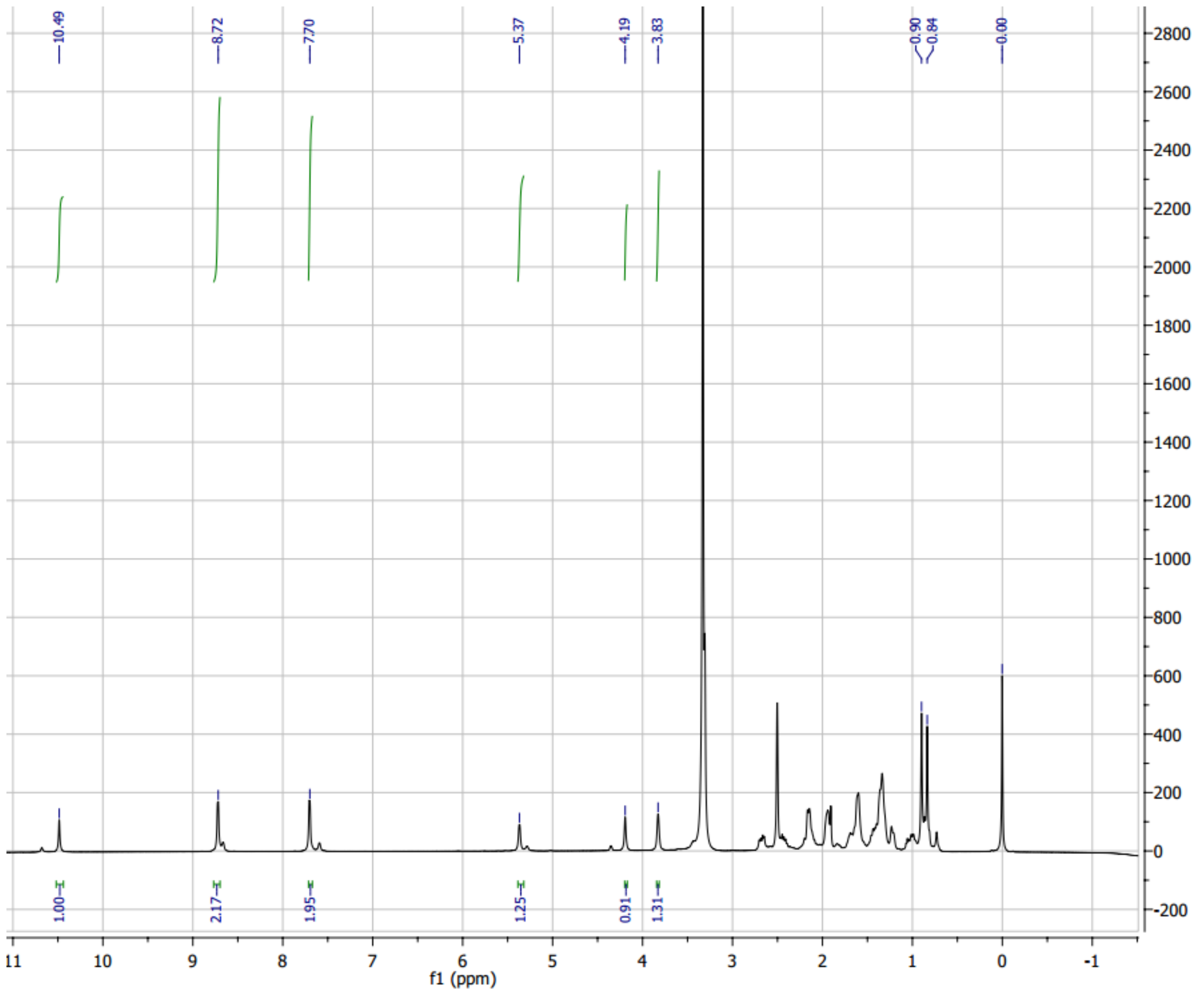

Figure S12: ^{1}H NMR spectrum of 3α-hydroxy-5α-androst-9(11)-en-17-one isonicotinoylhydrazone (compound **7**) in d_6-DMSO

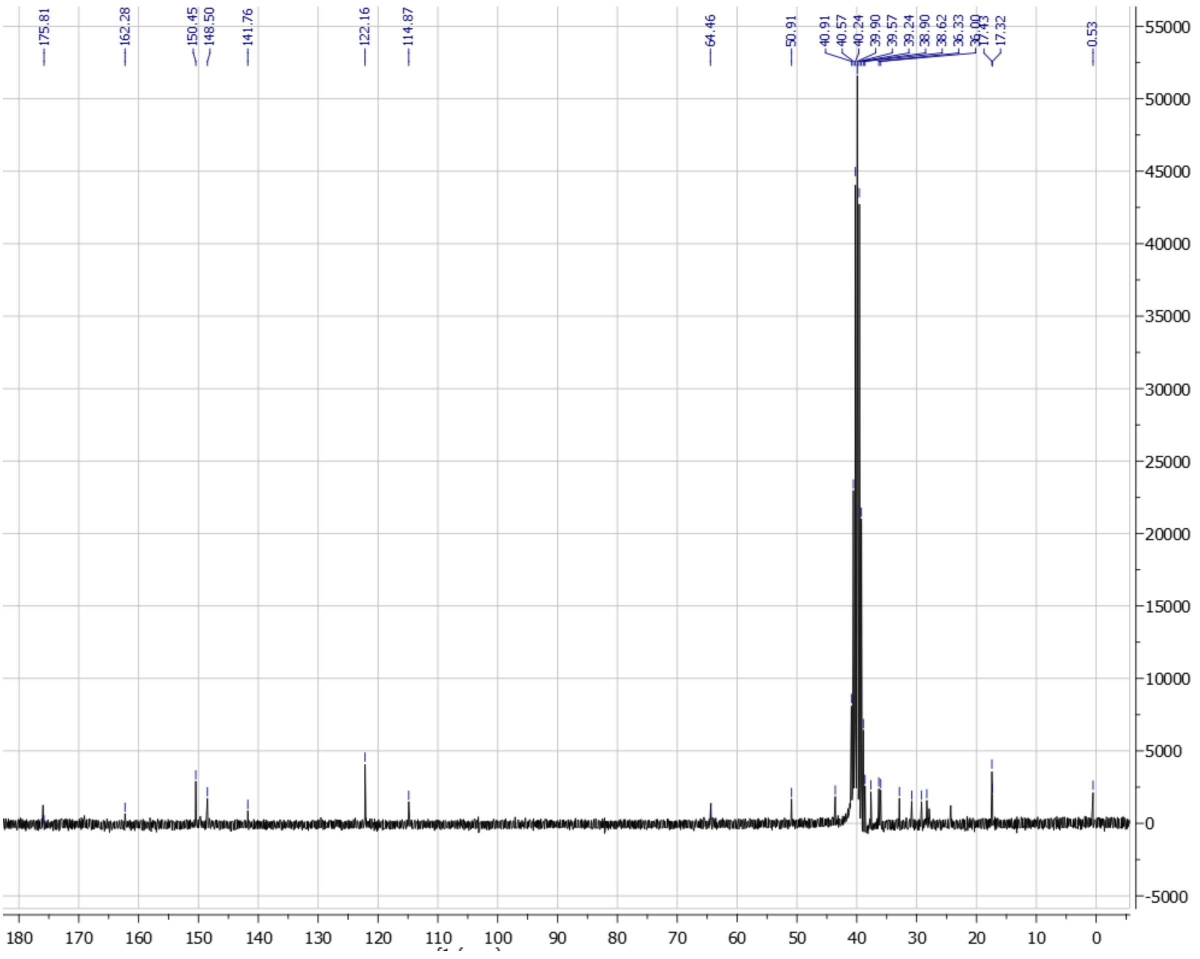

Figure S13: ^{13}C NMR spectrum of 3α-hydroxy-5α-androst-9(11)-en-17-one isonicotinoylhydrazone (compound **7**) in d_6-DMSO

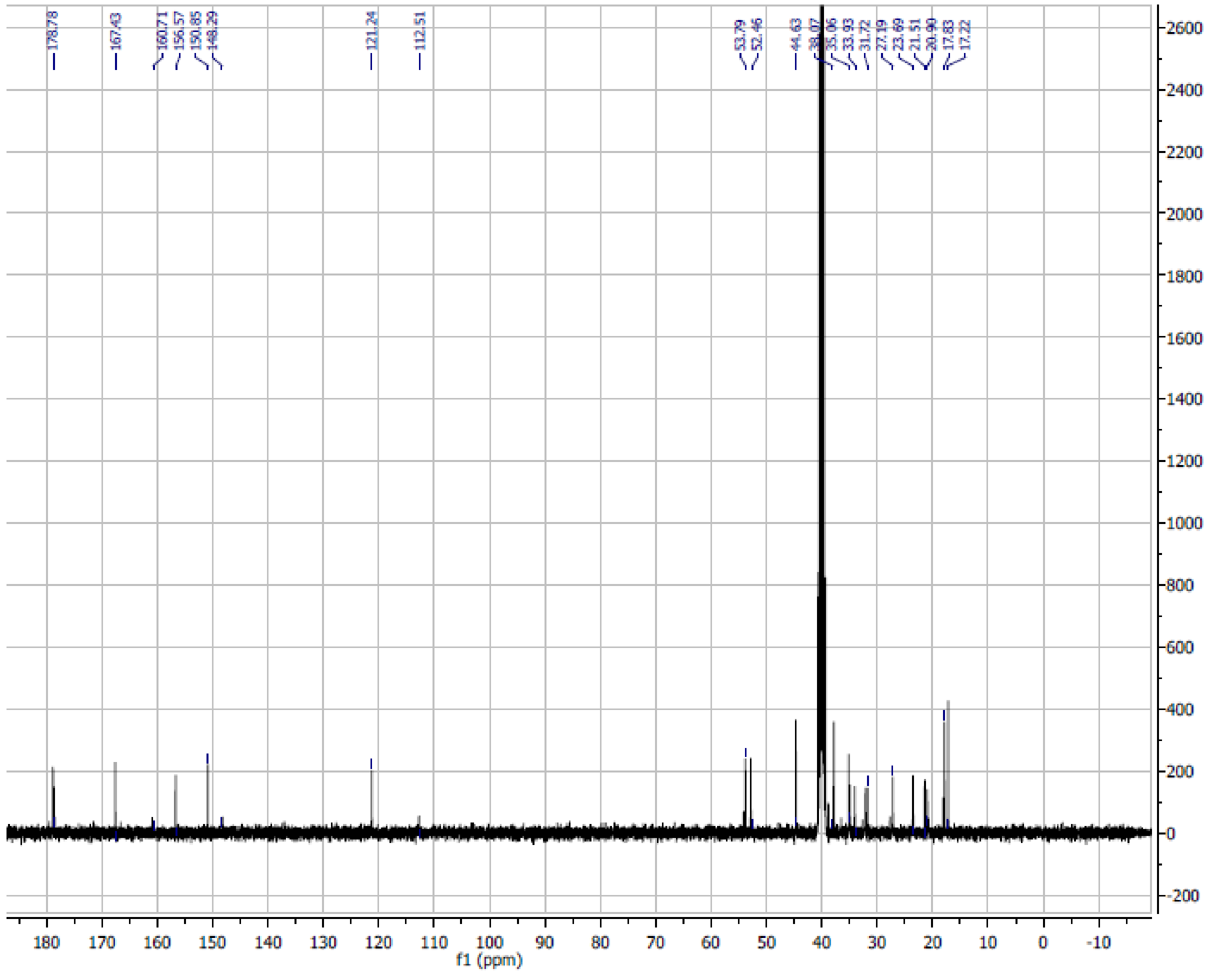

Figure S14: ^{13}C NMR spectrum of androsta-1,4-diene-3,17-dione dithiosemicarbazone (compound **8**) in d_6-DMSO

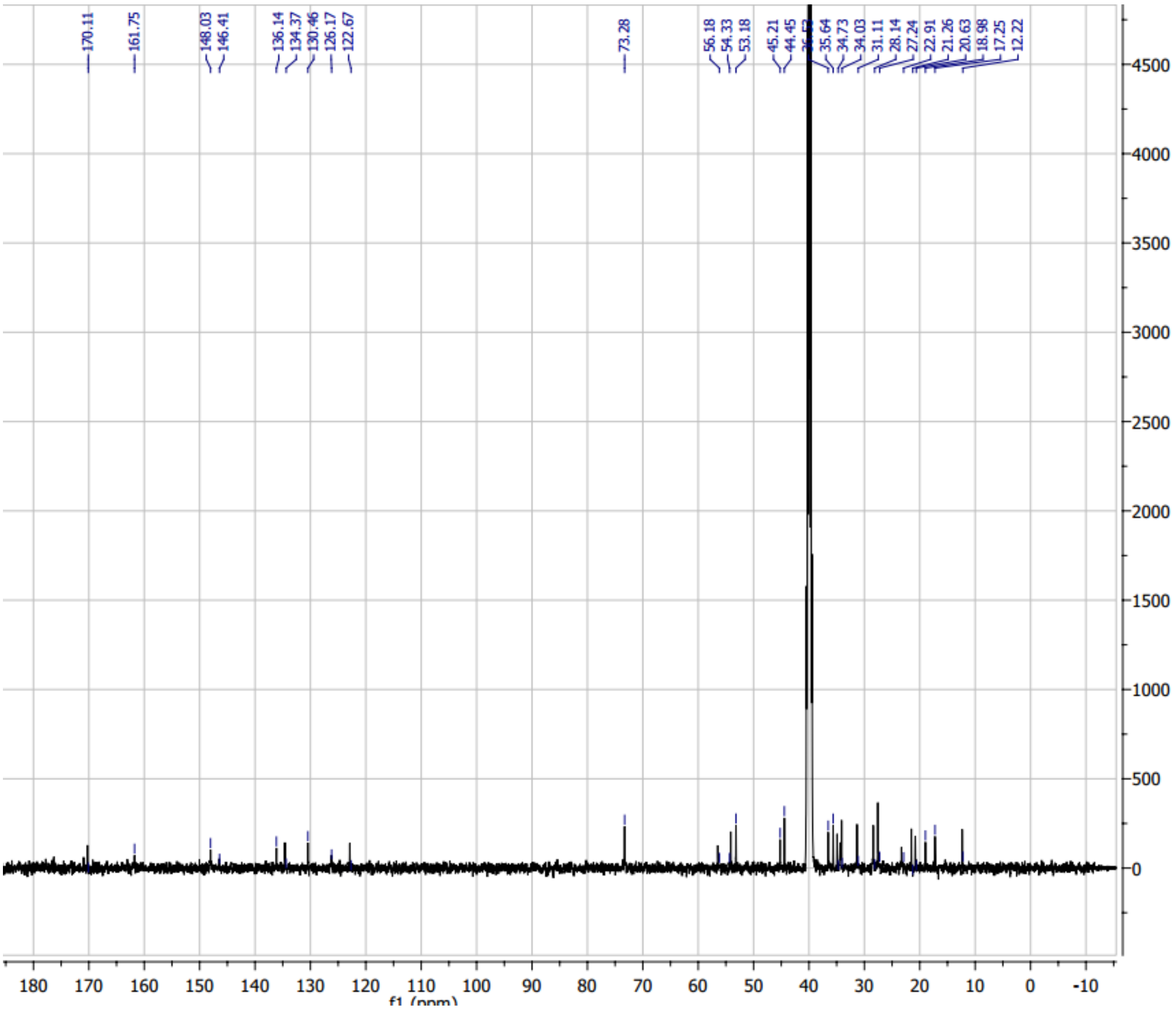

Figure S15: ^{13}C NMR spectrum of 3β-acetoxy-5α-androstan-17-one *m*-nitrobenzoylhydrazone (compound **9**) in d_6-DMSO

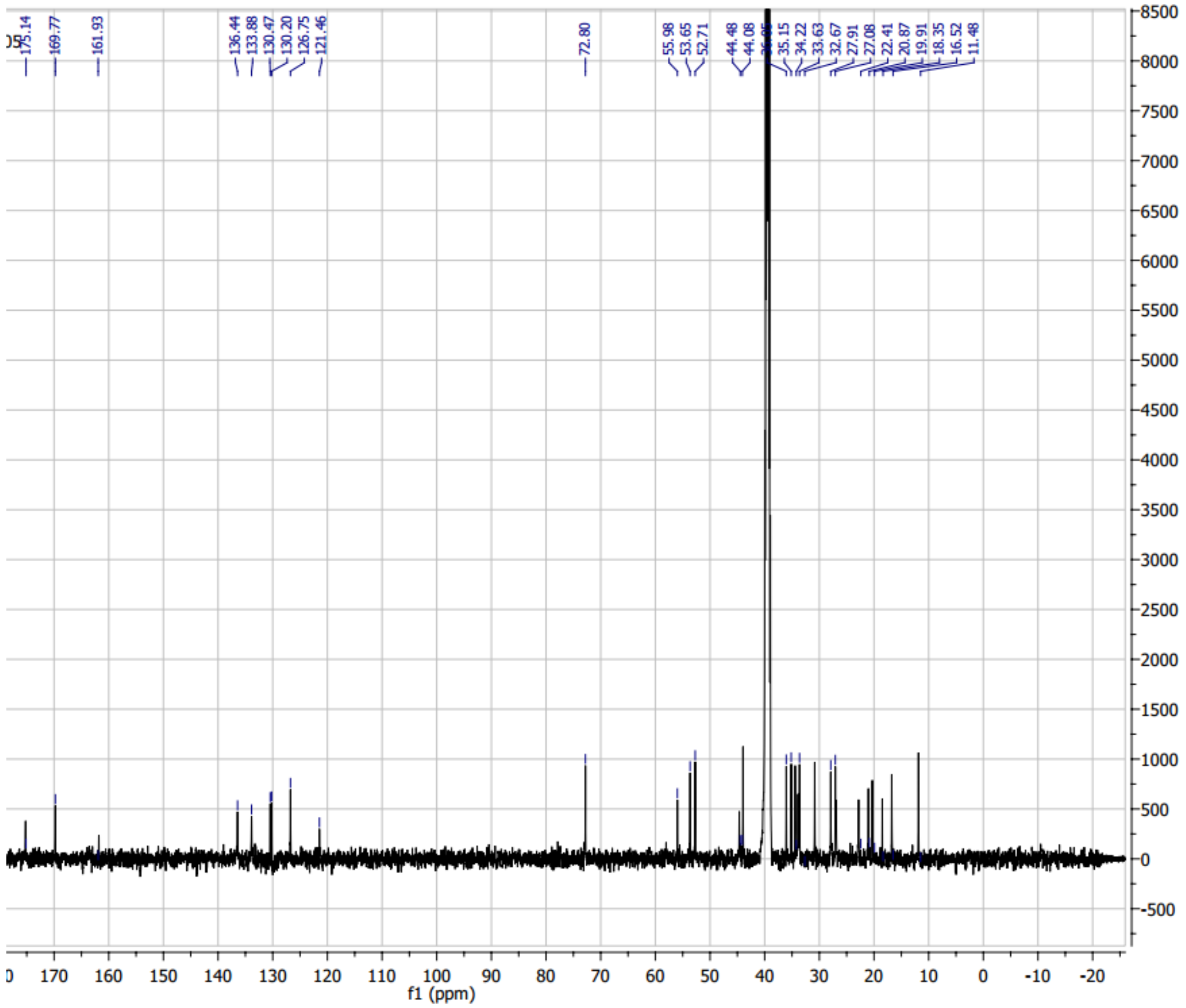

Figure S16: ^{13}C NMR spectrum of 3β -acetoxy-5α-androstan-17-one *m*-bromobenzoylhydrazone (compound **10**) in d_6-DMSO

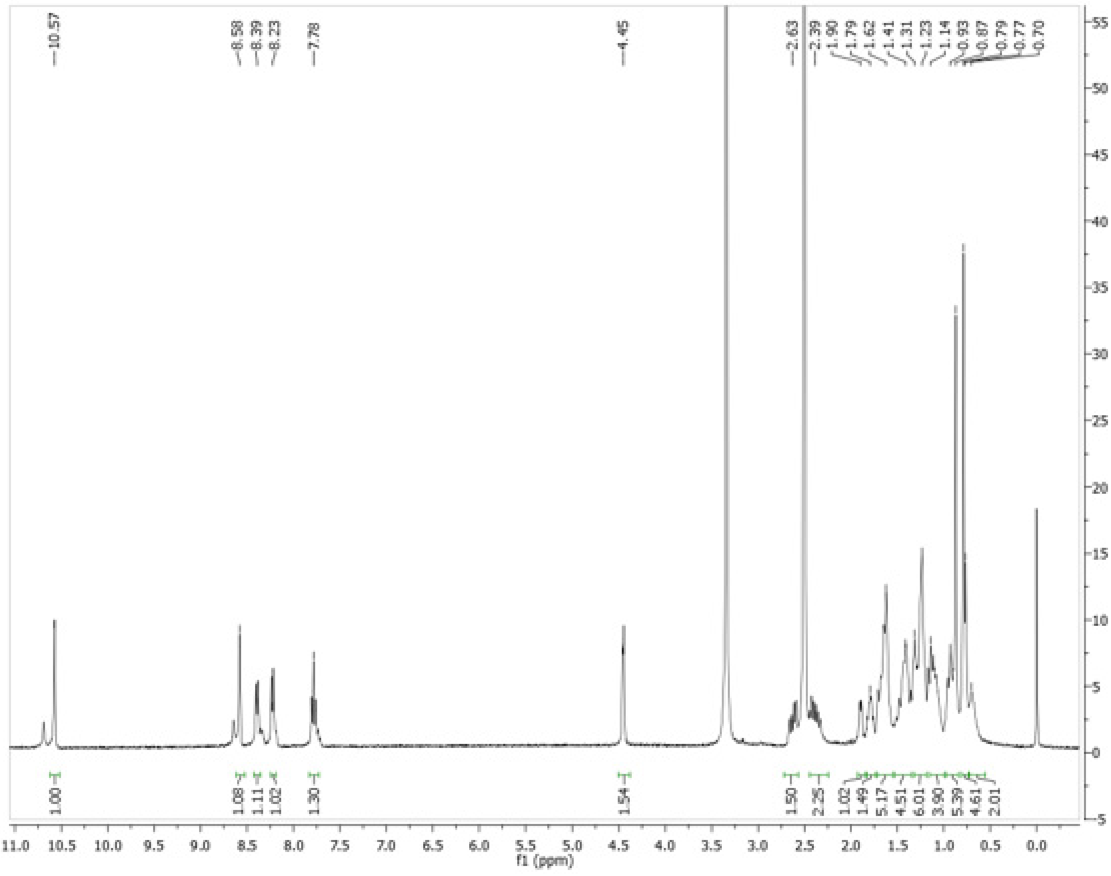

Figure S17: ^{1}H NMR spectrum of 3α -hydroxy-5α-androstan-17-one *m*-nitrobenzoylhydrazone (compound **11**) in d_6-DMSO

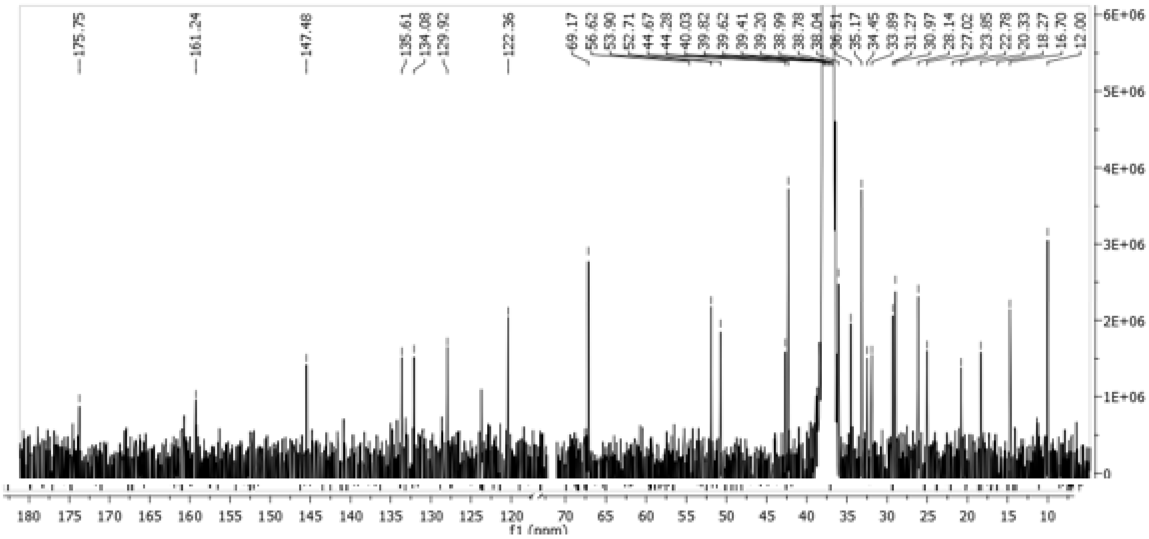

Figure S18: ^{13}C NMR spectrum of 3α -hydroxy-5α-androstan-17-one *m*-nitrobenzoylhydrazone (compound **11**) in d_6-DMSO

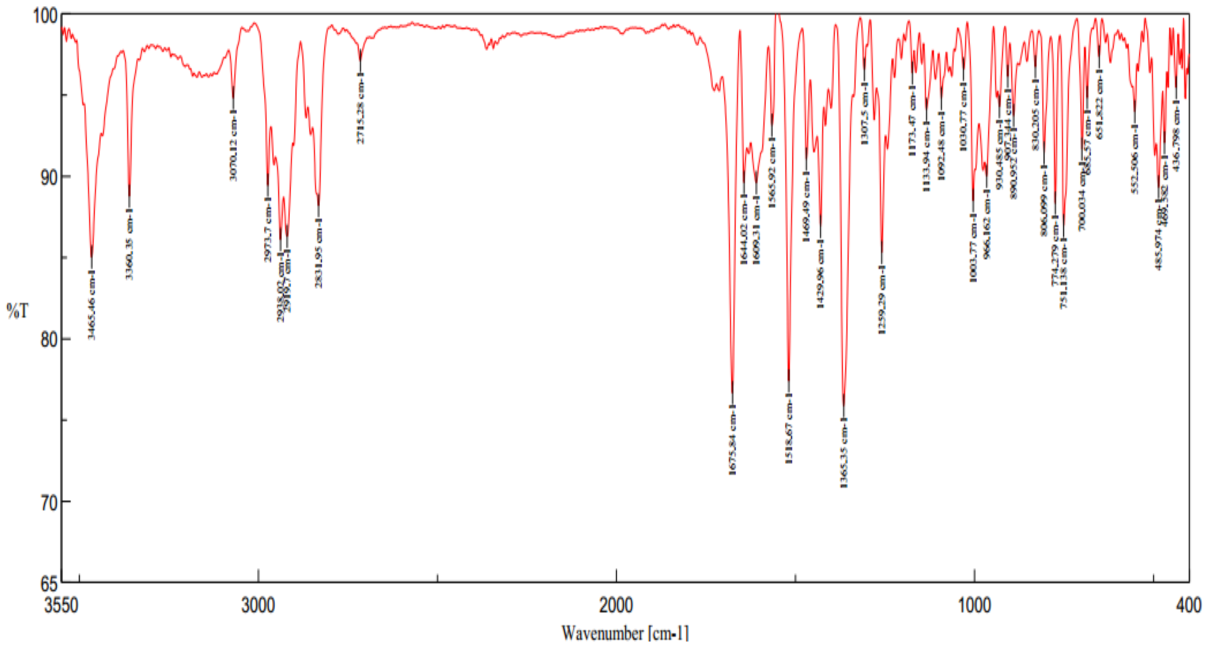

Figure S19: IR spectrum of 3α-hydroxy-5α-androstan-17-one *m*-bromobenzoylhydrazone (compound **12**) in KBr

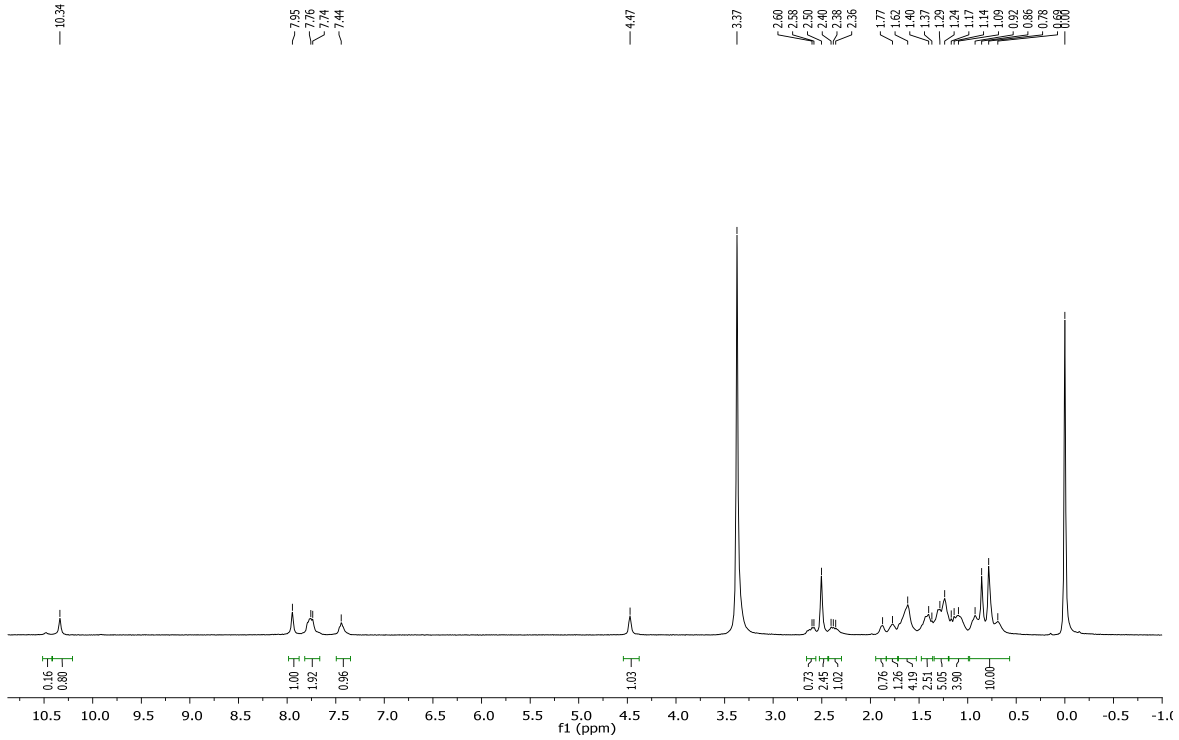

Figure S20: ^{1}H NMR spectrum of 3α-hydroxy-5α-androstan-17-one *m*-bromobenzoylhydrazone (compound **12**) in d_6-DMSO

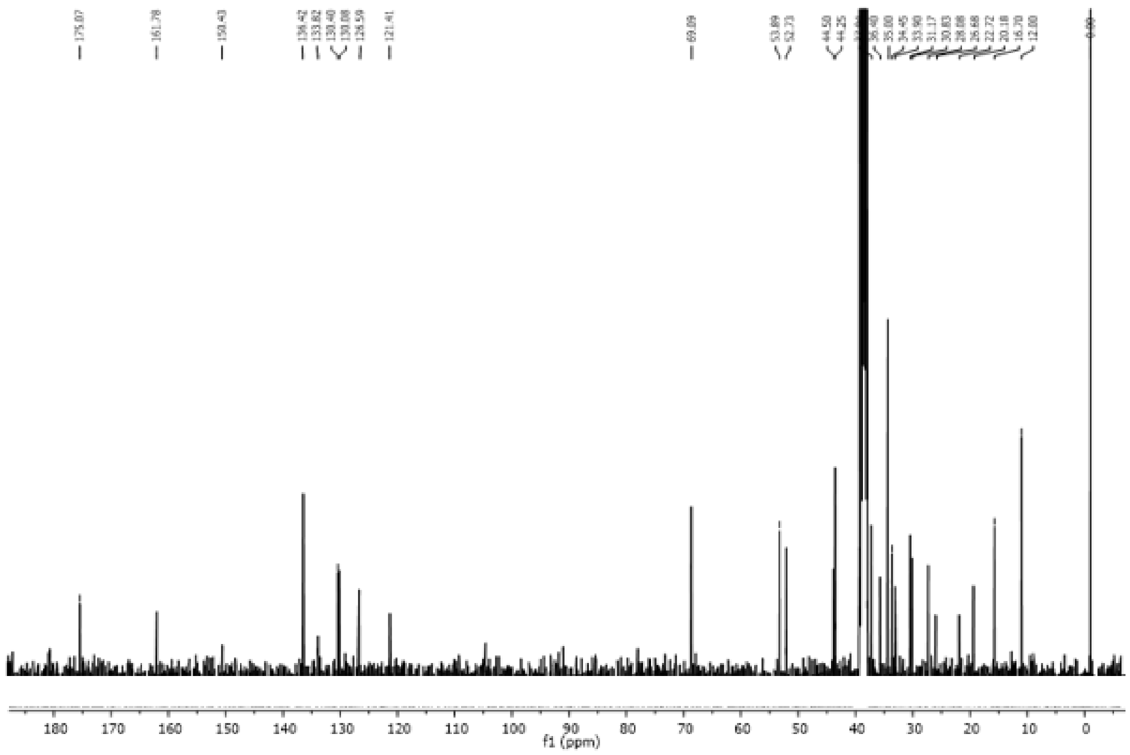

Figure S21: ^{13}C NMR spectrum of 3α-hydroxy-5α-androstan-17-one *m*-bromobenzoylhydrazone (compound **12**) in d_6-DMSO

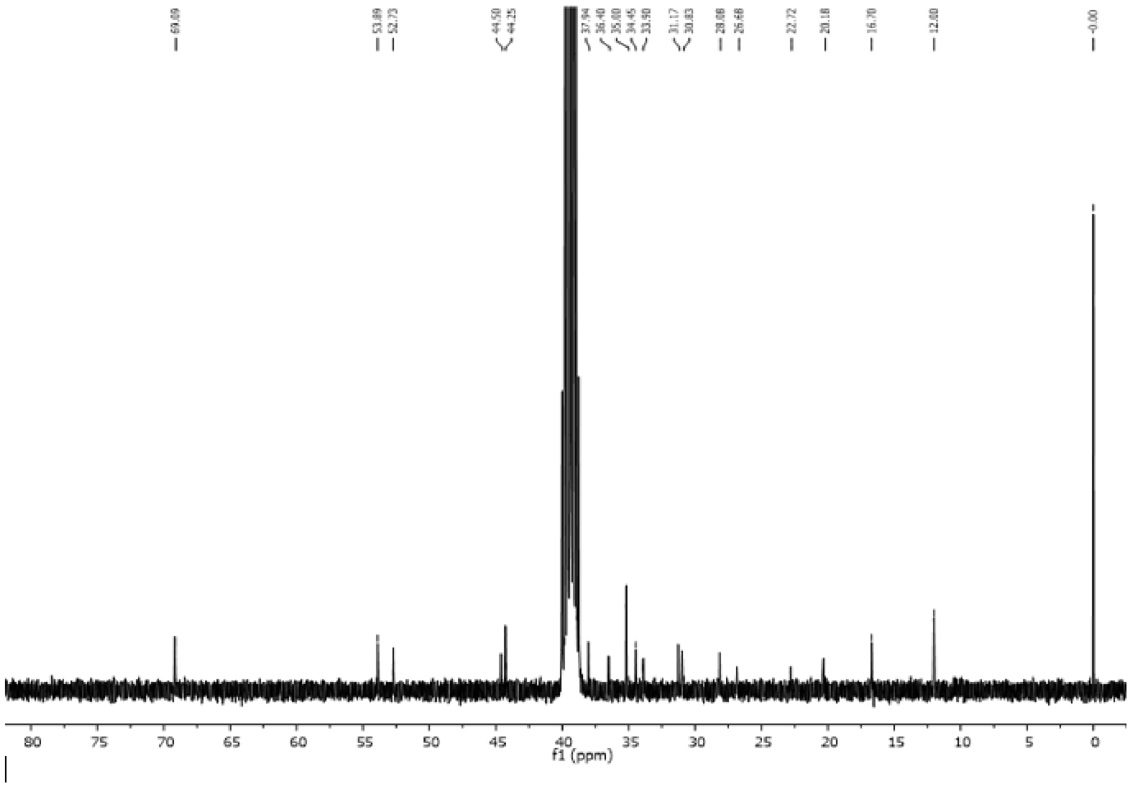

Figure S22: Fragment of ^{13}C NMR spectrum 3α-hydroxy-5α-androstan-17-one *m*-bromobenzoylhydrazone (compound **12**) in d_6-DMSO

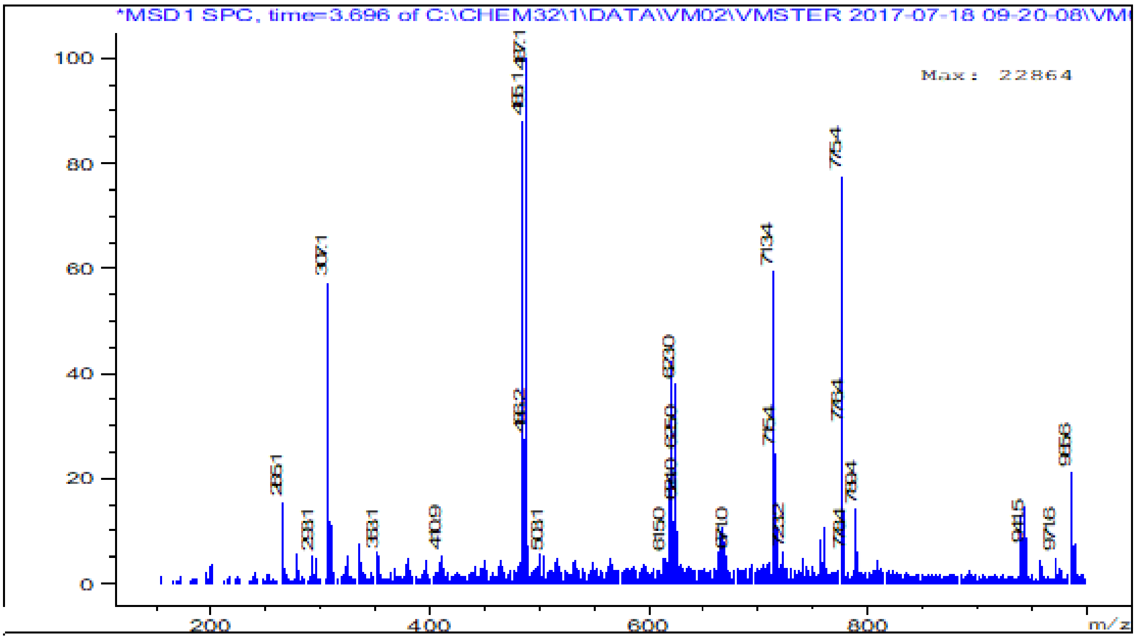

Figure S23: MS spectrum of 3α-hydroxy-5α-androstan-17-one *m*-bromobenzoylhydrazone (compound **12**)
